# Draft genome sequencing and secretome analysis of fungal phytopathogen *Ascochyta rabiei* provides insight into the necrotrophic effector repertoire

**DOI:** 10.1038/srep24638

**Published:** 2016-04-19

**Authors:** Sandhya Verma, Rajesh Kumar Gazara, Shadab Nizam, Sabiha Parween, Debasis Chattopadhyay, Praveen Kumar Verma

**Affiliations:** 1 National Institute of Plant Genome Research, Aruna Asaf Ali Marg, New Delhi-110067, India

## Abstract

Constant evolutionary pressure acting on pathogens refines their molecular strategies to attain successful pathogenesis. Recent studies have shown that pathogenicity mechanisms of necrotrophic fungi are far more intricate than earlier evaluated. However, only a few studies have explored necrotrophic fungal pathogens. *Ascochyta rabiei* is a necrotrophic fungus that causes devastating blight disease of chickpea (*Cicer arietinum*). Here, we report a 34.6 megabase draft genome assembly of *A. rabiei*. The genome assembly covered more than 99% of the gene space and 4,259 simple sequence repeats were identified in the assembly. A total of 10,596 high confidence protein-coding genes were predicted which includes a large and diverse inventory of secretory proteins, transporters and primary and secondary metabolism enzymes reflecting the necrotrophic lifestyle of *A. rabiei*. A wide range of genes encoding carbohydrate-active enzymes capable for degradation of complex polysaccharides were also identified. Comprehensive analysis predicted a set of 758 secretory proteins including both classical and non-classical secreted proteins. Several of these predicted secretory proteins showed high cysteine content and numerous tandem repeats. Together, our analyses would broadly expand our knowledge and offer insights into the pathogenesis and necrotrophic lifestyle of fungal phytopathogens.

Chickpea (*Cicer arietinum* L.), an important high-protein source, is an annual legume crop grown worldwide. The chickpea crop yield suffers primarily from Ascochyta blight (AB) that is caused by the necrotrophic ascomycete fungus *Ascochyta rabiei* (Pass.) Labr. [teleomorph: *Didymella rabiei* (Kovatsch.) Arx], causing up to 100% yield loss^1^. This directly penetrating fungus infects all the aerial parts of chickpea and produces several phytotoxins such as, solanapyrones A, B, and C; cytochalasin D; and a proteinaceous toxin^2^. The nature and degree of pathogenic variability in *A. rabiei* is still not clearly understood ever after several pathological and molecular studies. Therefore, comprehensive information on the biology and survival of *A. rabiei* is a prerequisite to develop more effective disease management strategies.

Fungal phytopathogens have adopted diverse lifestyles as a part of their infection strategies. Biotrophic pathogens have developed complex mechanisms and feeding structures to derive nutrition from their host plant while keeping them alive. In contrast, necrotrophic pathogens have developed mechanisms to kill their host swiftly to feed themselves and complete their lifecycle. Previously, necrotrophs were assumed to largely depend on the secretion of lytic and cell wall-degrading enzymes to damage the host tissue. However, recently it has been found that few necrotrophic fungi exploit the cell death machinery of the host plant instead of merely relying on lytic enzymes^3^,^4^. From this perspective, pathogen-encoded secreted proteins known as effectors play crucial roles in evading or suppressing the plant defense system. Most of the effectors have been characterized in biotrophic fungi and oomycetes. Nevertheless, the knowledge regarding necrotrophic effectors and the mechanisms by which they manipulate the host cell machinery remains limited, although initial establishment on the host is a prerequisite even for necrotrophs. Depending on the lifestyle of pathogens and host range, the effector repertoires diverge considerably. In order to acquire in-depth understanding of pathogenesis by necrotrophic fungi and infection mechanisms, the necrotrophy-associated genomic characteristics and sequences of the effector repertoire needs to be analyzed.

With this objective, we described the *de novo* whole genome assembly and comprehensive analysis of the *A. rabiei* genome. A large set of genes putatively involved in the pathogen-host interaction and polysaccharide degradation machinery were identified. The Carbohydrate-Active enZymes (CAZymes) repertoire of *A. rabiei* was identified and an in-depth comparison was performed with CAZymes of other representative necrotrophic and biotrophic fungi. The effector repertoire of *A. rabiei* was predicted and secretome annotation was performed to gain maximum knowledge about effectors related to necrotrophy. The predicted effector proteins of *A. rabiei* would be useful in identifying their resistant counterparts in chickpea and for designing efficient strategies against this devastating but poorly characterized pathogen.

## Results

### Genome sequencing, assembly and annotation

**Figure 1:**
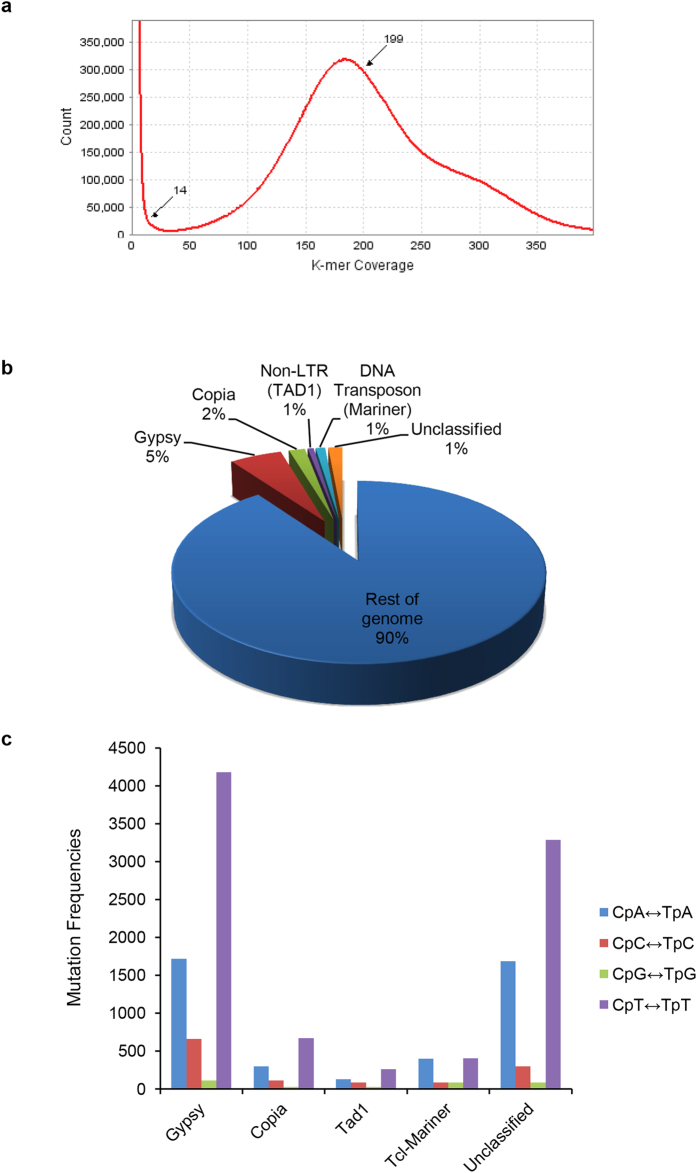
The *k*-mer distribution, repetitive sequences and repeat-induced point mutations in repetitive sequences of *A. rabiei*. (**a**) *K*-mer depth distribution plot of whole-genome Illumina reads. Paired-end reads of 64-mer were mapped to the genome using ABySS. A peak was identified at 178. **(b)** The percentage distribution of different types of repetitive elements in the *A. rabiei* genome. **(c)** The frequencies of all four types of di-nucleotide RIP mutations in different families of repeat elements are shown.

We used the genome of an Indian isolate of *A. rabiei* (ITCC No. 4638) for sequencing. This isolate was identified as mating type 2 (MAT1–2) using MAT locus-specific primers (Supplementary Fig. 1). Four paired-end libraries with average insert sizes ranging from 200 bp to 500 bp and a mate-pair library of 5 kb average insert size were sequenced using Illumina HiSeq1000 platform to generate 100 bp X 2 short sequence reads. Filtered high quality paired-end reads amounting to approximately 100 Gb sequencing data (Supplementary Tables 1,2) were assembled using ABySS^5^, resulting in a total assembly size of 34.6 Mb that is in accordance to estimated genome size of 23–34 Mb of 112 distinct *A. rabiei* isolates^6^,^7^. High coverage of 178X was achieved as estimated by *k*-mer analysis of read count versus *k*-mer coverage (Fig. 1a) and consisted of 338 scaffolds (Table 1), with N50 scaffold size of approximately 154.8 kb (Supplementary Table 2).

In total, 10,596 protein-coding genes were predicted from genome assembly using various *ab initio* gene prediction programs and open reading frames were validated by mapping unique expressed sequence tags, reported by Fondevilla *et al*.^8^, on the genome (Supplementary Data 1). Out of 128 cDNA sequences reported earlier in a transcriptome analysis of *A. rabiei* under oxidative stress^9^, 118 cDNA sequences were identified by BLAST search against assembled genome (E-value ≤ 1e-5). Gene Ontology (GO) terms were assigned to 5,511 protein-coding genes of *A. rabiei* (Supplementary Figs 2,3). Additionally, a total of 3,424 genes belonging to 327 pathways were annotated using the KEGG database (Supplementary Fig. 4, Supplementary Table 3, Supplementary Data 2, 3). The entire coding regions of the predicted genes constitute almost 47.6% of the genome. To evaluate the genome completeness of *A. rabiei*, a blast search was performed with highly conserved core eukaryotic genes^10^. Of the known 246 single-copy orthologs obtained from 21 fungal genomes, 245 are present in the *A. rabiei* genome. In addition, 247 of 248 core eukaryotic genes (CEGs) were also identified, which indicates that the assembly covered more than 99% of the gene space. The average gene density in the *A. rabiei* genome was 305 genes per Mb (Table 1), which was comparable to the average gene density of other closely related *Dothideomycetes* fungi^7^. The average gene length was 1,726 bp and consisted of an average of 1,526 bp of coding region and 193 bp of non-coding region. The genes with ORF length between 801–1000 bp are the most abundant (Supplementary Fig. 5). The overall GC content of the genome is 51.60%, whereas the GC content of the coding sequences and repetitive elements are 56.49% and 52.87%, respectively. Additionally, 125 tRNA genes were identified in the genome (Supplementary Table 4).

### Repetitive elements, repeat-induced point (RIP) mutations and simple sequence repeats (SSRs)

**Figure 2:**
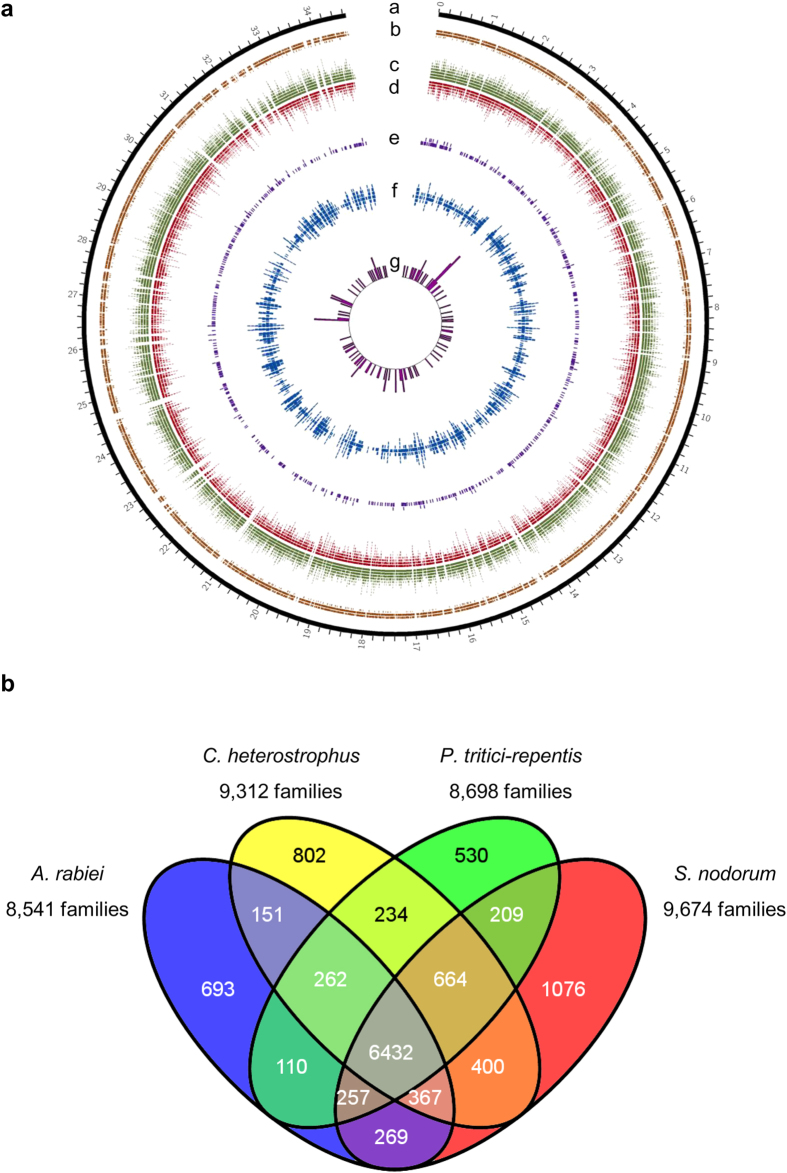
Circular map displaying genomic features of the *A. rabiei* genome and a comparison of orthologous genes. (**a**) Schematic representation of genomic characteristics of *A. rabiei* pseudo-genome (Mb scale). Circle a: pseudo-genome of 34.6 Mb. Circle b: positions of the protein coding genes. Circle c: positions of exons of the respective protein coding genes in circle b. Circle d: positions of introns of the respective protein coding genes in circle b. Circle e: distribution of the putative secretory proteins. Circle f: distribution of repetitive sequences in the *A. rabiei* genome. Circle g: histogram shows the tRNA density as represented by the number of tRNAs in 100-kb non-overlapping windows. The peak of the histogram correlates with the tRNA density. The diagram was plotted using Circos. (**b**) Venn diagram showing unique and shared orthologous gene families between and among the four closely related *Dothideomycetes* fungi. The orthologous gene families among *A. rabiei*, *C. heterostrophus*, *P. tritici-repentis* and *S. nodorum* were identified using OrthoMCL. Comparison of the four species revealed 693 gene families unique to *A. rabiei*. However, *A. rabiei* shares 151, 110 and 269 gene families with *C. heterostrophus*, *P. tritici-repentis* and *S. nodorum*, respectively. Moreover, 6,432 gene families are orthologous in all the four fungi.

RepeatScout^11^ was used to identify 155 repetitive families of repetitive sequences in the *A. rabiei* genome. These 155 families represent approximately 9.94% of the total genome (Figs 1b and 2a). Classification of the transposable elements (TEs) was performed using TEclass^12^. Of 155 repetitive families, 38 families were of DNA transposons, 72 families of LTRs, 1 family of LINEs and 6 families of SINEs. Thirty-eight families did not show homology to any of the existing class of TEs and hence, categorized as unclassified (Supplementary Table 5). All the repeat sequence families were further annotated manually by TBLASTX search against the fungal RepBase library^13^. Overall, 155 families of TEs accounted for 4,477 elements covering 3,445,339 bp (Supplementary Tables 5 and 6). DNA transposons (Class I TEs) and retrotransposons (Class II TEs) were abundant in *A. rabiei* and covered 335,061 bp and 2,610,681 bp, respectively (Supplementary Fig. 6, Supplementary Table 6). The majority of these are LTR retrotransposons/*Gypsy* followed by *Copia* LTR retrotransposons.

A fungal-specific genome defense mechanism known as RIP plays a major role in avoiding the deleterious and undesirable effects of proliferating TEs^14^. In Pezizomycotina, RIP gives rise to multiple C-to-T transition mutations in the repetitive sequences with a minor preference for CpA to TpA dinucleotides^15^. The degree of RIP in all repeated DNA families of *A. rabiei* was identified and quantified using RIPCAL^16^ (Supplementary Figs 7–11). Comparison of repeat families of TEs revealed nucleotide substitutions primarily representing C-to-T (G-to-A) transitions, indicating the action of RIP on all TE families (Fig. 1c). Moreover, the presence of orthologous genes of *Neurospora crassa* involved in RIP supports an active RIP defense mechanism in *A. rabiei* (Supplementary Table 7). Interestingly, high incidences of less likely CpT to TpT mutations in almost all the classes of TEs were observed (Supplementary Figs 7–11). The *Gypsy* class of transposon was clearly the most affected (Fig. 1c). In many filamentous ascomycetes such as *N. crassa*^17^, *Podospora anserine*^18^, *M. grisea*^19^, *Leptosphaeria* maculans^20^ and *Nectria* haematococca^21^, the CpA ↔ TpA mutation was experimentally demonstrated as preferred, leading to methylation of the sequences altered by RIP and resulting in effective silencing of the DNA sequences. For inactivation of TEs by RIP, nonsense mutations that are most effective would be generated most frequently by CpA ↔ TpA substitutions and never by CpT ↔ TpT substitutions. Therefore, a relatively lower frequency of CpA ↔ TpA mutations within the tandem repeats of the *A. rabiei* genome indicates RIP resistance in TEs. Another major regulatory mechanism to control gene expression in eukaryotes is RNA silencing^22^. The *A. rabiei* genome included key RNA silencing pathway genes such as RNA-dependent RNA polymerases, Argonaute-like proteins, RecQ family helicase and Dicer genes (Supplementary Table 7).

SSRs or microsatellites create and maintain genetic variations and play an active role in genome evolution^23^. However, little is known regarding SSRs in fungi. Therefore, a high-throughput SSR search to identify mono- to hexanucleotide SSR motifs in the *A. rabiei* genome was performed. In total, 4,259 SSRs, including 615 compound SSRs were identified in 307 scaffolds (Supplementary Table 8). Relative number of the SSRs was 123.09/Mb with 2,875.02 bp/Mb coverage. Trinucleotide SSRs were the most common SSR type in the genome assembly with 1,571 in number, representing nearly 36% of all SSRs (Supplementary Fig. 12a, Supplementary Table 9). They showed the highest relative abundance and relative density (Supplementary Fig. 12b,c). Of all compound SSRs, 432 interrupted SSRs (C) constituted 96.4% of the compound SSRs. In contrast, only 16 uninterrupted SSRs (C*) were found (Supplementary Table 10). The dinucleotide AG repeats were found to be the predominant, followed by trinucleotide CAC repeats (Supplementary Table 11). The SSRs, specifically the most abundant repeats, are known to have potential in contributing to the evolution of the genome. However, different fungal species have their own specific profile for SSRs type, abundance, occurrence and motif, which is independent of their genome sizes^24^.

### Comparisons with other fungal genomes

**Figure 3:**
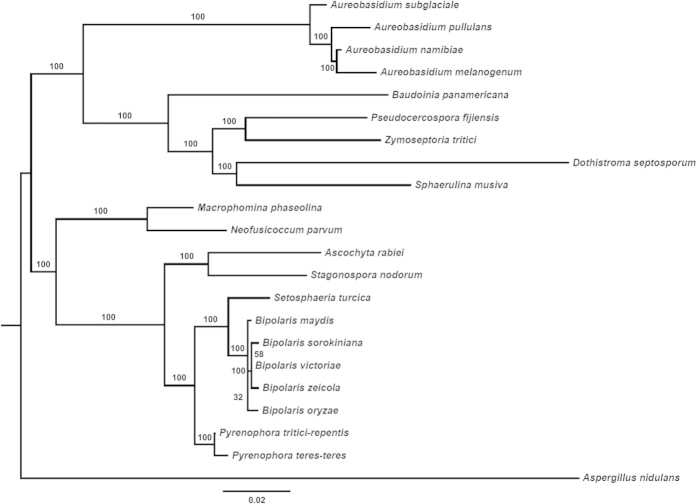
Phylogeny of selected *Dothideomycetes*. Estimated phylogenetic relationship of *A. rabiei* with other *Dothideomycetes* based on sequences of four protein-coding genes. Bootstrap based branch supports are shown, calculated according to the approximate Likelihood-Ratio Test, as implemented in RAxML 7.2.

The predicted proteome of *A. rabiei* was compared with a few closely related *Dothideomycetes*, i.e., *Cochliobolus heterostrophus*, *Pyrenophora tritici-repentis* and *Stagonospora nodorum*. OrthoMCL analysis showed that 6,432 (60.7%) of *A. rabiei* predicted proteins had orthologs in these three fungal species, while 693 (6.5%) predicted proteins were unique (Fig. 2b). Interestingly, 693 unique proteins were predicted to encode 53 glycoside hydrolases (GHs) (Supplementary Fig. S13, Supplementary Table 12). A large number of predicted proteins exhibited very high sequence similarity with those of necrotrophic wheat pathogen *S. nodorum* (6,701, 63.2%), indicating it as the nearest species among the selected fungi (Supplementary Data 4). Phylogenetic analysis of *A. rabiei* along with other 21 selected fungal species (20 *Dothideomycetes* and one *Eurotiomycetes* outgroup) also suggested that *A. rabiei* was closely related to *S. nodorum* (Fig. 3).

Pfam annotation was assigned to 7,118 genes (67.17%) (Supplementary Data 5). The Pfam domains identified within the *A. rabiei* proteome were compared with those present in the three most closely related *Dothideomycetes* fungi (Supplementary Data 6). The *A. rabiei* proteome unveiled high abundance of major facilitator superfamily (MFS) transporters, protein kinases, short-chain dehydrogenases/reductase family, zinc cluster domains and sugar transporters. In contrast, cytochrome P450 family proteins were significantly less abundant in *A. rabiei*. Moreover, unlike *C. heterostrophus* and *S. nodorum* but similar to *P. tritici-repentis*, heterokaryon incompatibility protein (HET) in *A. rabei* was found in low abundance. The protein-protein interaction (WD40, PF00400) and FAD binding domains (PF01565) were found considerably less in *A. rabiei* than in the other three fungi, suggesting variation in *A. rabiei* genome from its closely related *Dothideomycetes* fungi.

**Figure 4:**
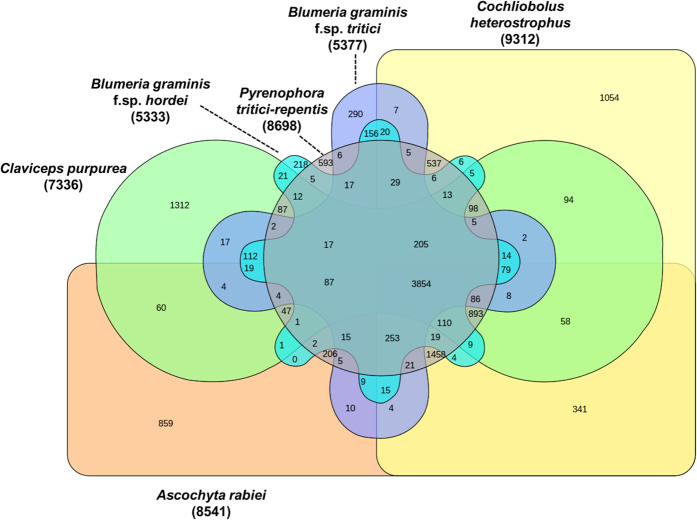
Comparison of orthologous genes between necrotrophic and biotrophic fungi. Venn diagram showing the distribution of unique and shared orthologous gene families between and among the three necrotrophic and three biotrophic ascomycete fungi based on gene family cluster analysis. The orthologous gene families among *A. rabiei*, *C. heterostrophus*, *P. tritici-repentis*, *Blumeria graminis* f.sp. *tritici*, *Blumeria graminis* f.sp. *hordei* and *Claviceps purpurea* were identified using OrthoMCL. Comparison revealed 1,458 genes are orthologous in the selected three necrotrophic fungi. Additionally, 112 orthologous genes are present among the selected three biotrophic fungi.

Comparative analysis was carried out between a set of necrotrophic (*A. rabiei*, *C. heterostrophus* and *P. tritici-repentis*) and biotrophic fungi (*Blumeria graminis* f.sp. *tritici*, *Blumeria graminis* f.sp. *hordei* and *Claviceps purpurea*). OrthoMCL analysis showed that 1,458 and 112 proteins were orthologous among the necrotrophic and biotrophic fungi, respectively (Fig. 4). Annotation of 1,458 orthologous proteins showed that most of them were transporters and enzymes (Supplementary Data 7) whereas, 112 orthologous proteins were mostly related to cell fusion, morphogenesis, voltage gated calcium channels and DNA damage repair (Supplementary Data 8). Interestingly, 1,458 orthologous proteins among necrotrophs had 296 CAZymes (20%) whereas 112 orthologous proteins among biootrophs had only 13 CAZymes (11%) (Supplementary Table 13,14).

### Transporters and secondary metabolism regulation

Transporters involved in nutrient uptake and re-allocation play multiple vital roles in growth and development. In total, 821 transporter proteins belonging to 90 families were identified in *A. rabiei* assembly (Supplementary Table 15). The highest number of transporters belonged to electrochemical potential-driven transporters superfamily (368), followed by primary active transporters superfamily (265) (Supplementary Fig. 14). Among all the transporters, the MFS transporters (165), which are involved in secondary metabolism, were the most abundant. In addition, 55 alpha-type channels and 52 ATP-binding cassette (ABC) transporters were also identified. The alpha-type channels facilitate energy-independent diffusion while ABC transporters participate in polysaccharide, lipid and amino acid transport. The abundance of MFS transporters and the presence of ortholog of *Saccharomyces cerevisiae* GPR1 (the glucose or sucrose sensing receptor) in the genome suggested that *A. rabiei* might possess a broad specificity for utilizing nutrients from host plants.

Biosynthesis of secondary metabolites, such as mycotoxins, alkaloids and pigments in response to environmental conditions is vital for fungal development. In the *A. rabiei* genome assembly, 26 clusters harboring putative secondary metabolite genes were identified, suggesting possible production of biologically active compounds (Supplementary Table 16). Nine T1 polyketide synthase (T1PKS) gene clusters were present, in contrast to only one T3PKS gene cluster. These PKS genes lied within the clusters of genes encoding dehydrogenases, oxidoreductases, methyltransferases and cytochromes P450, which are responsible for modifying secondary metabolites. Further, only two non-ribosomal peptide synthetase (NRPS) gene clusters were identified that harbored FAD-dependent oxidoreductases and monooxygenases. In addition, six gene clusters of terpenes required for producing mycotoxins are also present. Furthermore, the genes involved in cytochalasin toxin production were identified in *A. rabiei* genome. The cytochalasin gene cluster consisting of 14 genes is reported in *Aspergillus clavatus* genome^25^. Out of those, orthologs of 11 genes were identified in *A. rabiei* genome assembly (Supplementary Data 9). Therefore, the *A. rabiei* genome represents rich resources for secondary metabolite biosynthesis that may be responsible for the production of several secondary metabolites such as, mycotoxins, alkaloids and pigments.

### Polysaccharide degradation machinery and gene families involved in pathogenicity

**Figure 5:**
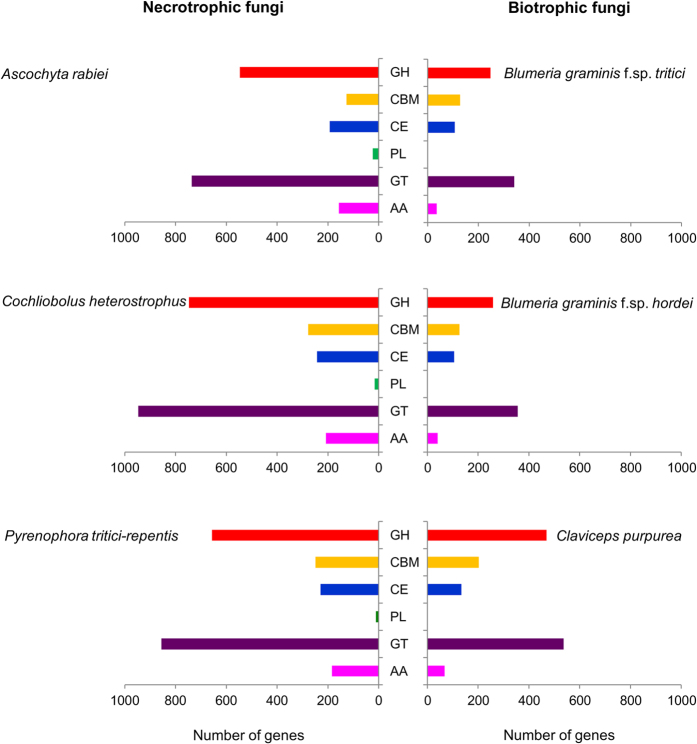
Diversity of genes encoding Carbohydrate Active enZymes (CAZymes). The bars represent the number of genes dedicated to CAZymes in the genomes of six fungi. Necrotrophic fungi: *A. rabiei*, *C. heterostrophus* and *P. tritici-repentis* are shown on the left; Biotrophic fungi: *Blumeria graminis* f.sp. *tritici*, *Blumeria graminis* f.sp. *hordei* and *Claviceps purpurea* are shown on the right. All six CAZyme categories are represented: carbohydrate-binding modules (CBMs), carbohydrate esterases (CEs), glucoside hydrolases (GHs), glycosyl transferases (GTs), polysaccharide lyases (PLs) and auxiliary activities (AAs).

Enzymes required for degrading plant cell walls is a crucial factor for pathogen invasion. Not surprisingly, the growth efficiency and aggressiveness of phytopathogens are often associated with their CAZymes. Among the 10,596 unique proteins of *A. rabiei*, 1,727 (16.3%) showed presence of Pfam protein domain that matched with at least one of the CAZyme families. These putative CAZymes included 58 families of glycoside hydrolases (GHs), 40 families of glycosyl transferases (GTs), 8 families of carbohydrate esterases (CEs), 18 families of carbohydrate-binding modules (CBMs), 9 families of auxiliary activities (AAs) and only 3 families of polysaccharide lyases (PLs) (Supplementary Fig. 15a, Supplementary Table 17). Among all the CAZyme families, the GT family is the most represented, followed by the GH proteins. The most abundant GT classes were strongly geared toward cellulose (GT48), hemicellulose (GT34) and chitin (GT2) degradation (Supplementary Fig. 15b, Supplementary Table 17). The relationship between the number and variety of CAZymes, and fungal nutritional strategy was examined by comparing predicted CAZymes of *A. rabiei* with those in few other related necrotrophic and biotrophic fungi. Unlike biotrophs, *A. rabiei* and other necrotrophic fungi had a significantly expanded set of CAZymes (Fig. 5), particularly cellulose and hemicellulose degrading enzymes (Supplementary Figs 16,17). Further study would be required to determine their relevance to plant pathogenicity or other lifestyle characteristics. However, these findings indicated that *A. rabiei* possessed a battery of CAZymes that would be suitable for the consumption of carbohydrates commonly found in plant hosts and also for the degradation of pectin.

To examine potential pathogenicity genes in *A. rabiei*, genome-wide BLAST analyses using the protein sequences in the Pathogen-Host Interaction Database (PHI database)^26^ were performed. In total, 2,707 protein-coding genes in *A. rabiei* were predicted to be orthologous to PHI genes (Supplementary Data 10), of which 1,444 (13.6%) genes were predicted to be involved in virulence and pathogenicity (Supplementary Fig. 18, Supplementary Table 18). GO in biological processes revealed that the majority of the protein-coding genes that were orthologous to PHI genes were associated with metabolic processes including degradation enzymes for large molecules, which might be involved in breaking host physical barriers (Supplementary Fig. 19). The genes associated with oxidoreductase activity were also highly abundant. Catalytic activity and binding activity were prevalent GO terms in molecular function (Supplementary Data 11), suggesting presence of an array of genes involved in pathogen-host interaction and the survival of *A. rabiei* during its life-cycle.

### Prediction and analysis of *A. rabiei* secretome

For successful infection, pathogenic fungi largely depend on an arsenal of secreted proteins, particularly effectors. A comprehensive pipeline was designed to carry out the prediction of *A. rabiei* secretome (Supplementary Fig. 20). *A. rabiei* genome encodes 758 potentially secreted proteins (7.1% of predicted proteins) including 538 classical and 220 non-classical secreted proteins. Interestingly, 52 classical and 20 non-classical secretory proteins were present among 693 proteins unique in *A. rabiei* (Fig. 2b, Supplementary Data 12). For predicting non-classical secreted proteins, SecretomeP v1.0^17^ was included in computational pipeline. For analyzing predicted *A. rabiei* secretome, GO terms were assigned to 354 putative secretory proteins in three GO categories: molecular function (334), biological process (321) and cellular component (71) (Supplementary Fig. 21a). Fifty-five genes that were common to all the three categories were identified (Supplementary Fig. 22). Under biological process, categories such as carbohydrate metabolic process, protein metabolic process, single-organism process, cellular metabolic process, response to oxidative stress and others were highly represented (Supplementary Fig. 21b). Within molecular function ontology, proteins associated with hydrolase activity, oxidoreductase activity and ion binding were most abundant. In the cellular component category, proteins for extracellular region, cell and membrane were highly abundant. These results indicated that the secretome of *A. rabiei* exhibits high metabolic activity and responds to oxidative stress encountered during host invasion.

**Figure 6:**
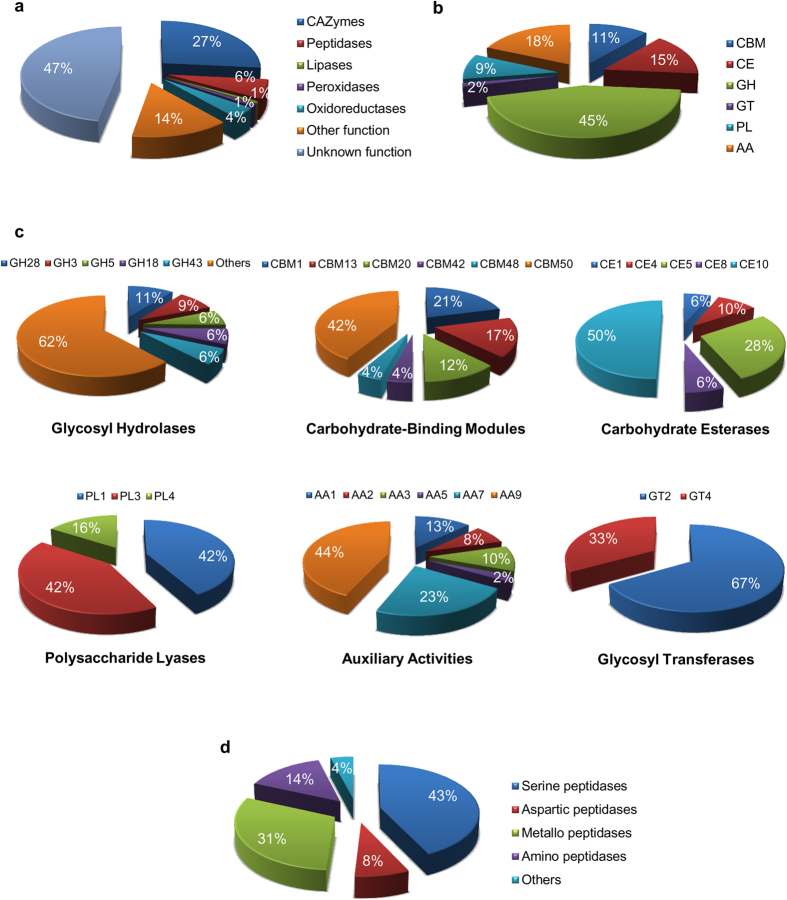
Functional annotation of the *A. rabiei* effector candidates. (**a**) Percentage distribution of the proteins with distinct enzymatic functions. (**b**) Summary of the six CAZyme categories: carbohydrate-binding modules (CBMs), carbohydrate esterases (CEs), glucoside hydrolases (GHs), glycosyl transferases (GTs), polysaccharide lyases (PLs) and auxiliary activities (AAs). **(c)** Distinct summaries of each of the CAZyme categories representing the most abundant CAZyme classes. The prediction of CAZymes from *A. rabiei* effector candidates and their classification were performed using tools from the Carbohydrate-Active EnZymes (CAZyme) database. (**d**) Percentage distribution of different types of peptidases present in *A. rabiei* secretome.

In addition, 201 effector candidates (26.5% of the total secretome) were annotated with the CAZyme database (Fig. 6a,b, Supplementary Table 19). The repertoire of secreted CAZymes consisted of 36 families of GHs, 2 families of GTs, 5 families of CEs, 3 families of PLs and 6 families each of CBMs and AAs. The 36 families of GHs comprising of 95 CAZymes was the most common (47%) in the total secreted CAZymes (Fig. 6a), followed by 6 families of AAs that contributed 19% to the overall secreted CAZymes. These analyses suggested existence of a clear dual preference in *A. rabiei* secreted CAZymes. Very high prevalence of GHs, CEs and AAs, which are required for degradation of the structures of plant cells was observed. In contrast, CBMs that functions in modification of the fungal cell wall for growth or protection from host-defenses were also in abundance. The most prevalent GHs CAZyme class was GH28 and GH43 which represented polygalacturonase and xylanase, respectively (Fig. 6c, Supplementary Table 19). Polygalacturonase and xylanase degrades polygalacturonan and hemicellulose, respectively, present in the plant cell walls to convert plant material into usable nutrients. The most abundant classes of CBMs were CBM50, CBM1 and CBM13 that consists of LysM domain containing proteins. The LysM domain-containing fungal effectors have been shown to inhibit plant chitinases^28^. Moreover, they bind to chitin to prevent elicitation of pathogen associated molecular pattern (PAMP) triggered immunity (PTI) and, thereby, prevent induction of host defense^29^. *A. rabiei* secretome also contained distinct peptidases, lipases, peroxidases and oxidoreductases (Fig. 6a,d). Therefore, these analyses suggested that the secretome of *A. rabiei* consists of proteins of diverse nature, which might function in facilitating proper colonization of the fungus, degradation of the host plant matter to acquire nutrients and inactivation of the host defenses.

**Figure 7:**
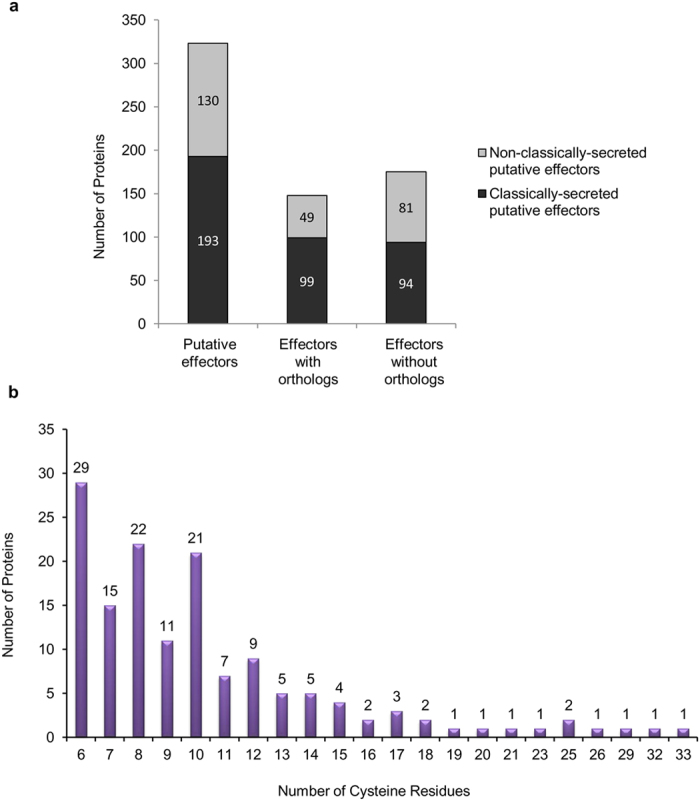
*A. rabiei* effector orthologs and summary of the cysteine-rich effector proteins. (**a**) Both classically and non-classically secreted A. rabiei putative effector candidates are shown. The number of effector candidates with and without orthologs in closely related *Dothideomycetes*, i.e., *C. heterostrophus*, *P. tritici-repentis*, and *S. nodorum* are shown. (**b**) The unannotated secretory proteins or those that could not be assigned any enzymatic function were analyzed for the presence of a high number of cysteine residues (≥ 6). The *x*-axis shows the total number of cysteine residues present in a protein sequence, and the *y*-axis denotes the number of proteins harboring these cysteine residues.

In order to determine conservation of *A. rabiei* putative effectors, 323 putative effectors (lacking CAZymes and known domain proteins) were searched for the presence of orthologs in closely related *C. heterostrophus*, *P. tritici-repentis*, and *S. nodorum* (Query coverage ≥50%, identity ≥40%). Out of 323, only 148 putative effectors had their orthologs in at least one of the three fungi whereas, 175 putative effectors were unique to *A. rabiei* suggesting that effectors are less conserved in nature (Fig. 7a, Supplementary Data 13). Moreover, particularly non-classically secreted effectors were found less conserved as compared to classically secreted effectors.

In total, 167 effector candidates were annotated using PHI database (Supplementary Fig. 23, Supplementary Table 20). The BLAST analyses predicted that 70 secretory proteins, accounting for 9.2% of the total secretome, were putatively involved in virulence and pathogenicity. Furthermore, the non-annotated 367 effector candidates were explored for the presence of high cysteine content (≥6) and multiple tandem repeats (≥9,) that are characteristic features of effector proteins. We identified a total of 145 proteins that contained 6 or more cysteines (Fig. 7b). In total, 21 predicted effector proteins had 9 or more tandem repeats in them (Supplementary Table 21). Further analysis predicted extracellular space as the *in planta* location for the majority of the mature effector candidates (Supplementary Table 22). Approximately 164 mature effector candidates was predicted for nuclear localization *in planta*, and among them, 18 showed the presence of a nuclear localization signal (Supplementary Table 23).

## Discussion

Necrotrophic fungi are drawing more attention due to their unique lifestyle and devastating nature. However, their strategies for pathogenesis are difficult to understand. The *A. rabiei*-chickpea system provides an excellent model for studying the mechanisms involved in the pathogenesis of such fungi. *A. rabiei* is an economically important pathogen of chickpea and genome sequence of chickpea is available^30^. In addition, this fungus is fast growing under laboratory conditions, and genetic manipulations/transformations are easy^31^. Necrotrophic fungi are generally resistant to a hypersensitive response, suggesting that they possess an inventory of effectors to counteract host-generated oxidative stress^9^,^32^,^33^ and to induce host cell death. However, recent evidences suggest that effectors play crucial role in suppressing the host defense, thus, making the initial events of necrotrophy similar to biotrophy^34^,^35^. In this study, we sequenced, assembled and analyzed the whole genome of *A. rabiei*. The total assembly size was 34.6 Mb, which was within the range of *Dothideomycetes* genomes (33.5–49 Mb)^7^. Both RNA silencing and RIP mechanisms act in *A. rabiei* to counteract the adverse effects of proliferating TEs. The higher occurrence of CpT ↔ TpT transitions and relatively lower frequency of CpA ↔ TpA mutations were observed as prominent features. However, CpT ↔ TpT substitutions do not code for nonsense mutations, suggesting RIP resistance in the tandem repeats. A similar phenomenon has been observed in *M. grisea* accompanying CpA-targeted mutation in RIP-affected sequences^19^.

The comparative genome analyses suggested maximum closeness of the *A. rabiei* genome to the necrotrophic wheat pathogen *S. nodorum* (Fig. 2b). In addition, the protein-coding genes in *A. rabiei* were relatively less in number (10,596) compared to those in the genomes of the necrotrophic fungal pathogens *P. tritici-repentis* (12,141)^36^ and *S. nodorum* (12,383)^37^ or the hemi-biotrophic *C. sativus* (12,250)^38^. This lower number may be due to presence of fewer genes in *A. rabiei* or a result of the stringent methodology of gene prediction adopted to minimize redundant genes. Particularly, four categories of functional proteins were drastically reduced in *A. rabiei*. First, the WD40, ankyrin repeat, BTB (for BR-C, ttk and bab) and other domains that are involved in protein-protein interactions were significantly fewer. This indicates that in *A. rabiei*, lower abundances of a few families of proteins involved in protein-protein interactions, scaffolding proteins and enzymes with varying co-factors suggested broader specificity of these families for their downstream proteins to perform necessary biological functions despite of their low abundance. Secondly, few classes of enzymes with varying co-factors (such as flavin adenine dinucleotide, adenosine monophosphate and nicotinamide adenine dinucleotide) were less in number and suggested that these enzymes classes might have broad specificity for their substrates in *A. rabiei* for carrying out important enzymatic reactions essential for its life cycle. Third, the HETs were also significantly fewer compared to those in *C. heterostrophus* and *S. nodorum* genomes. In filamentous fungi, genetic differences in HETs are known to limit viable heterokaryon formation between two different WT strains^39^. Low abundance of HETs in *A. rabiei* indicated a higher tendency to fuse with dissimilar WT strains, leading to a higher probability of horizontal transfer of genetic elements. Fourth, the CYPs that are involved in detoxification of phytoalexin repertoires of host plants^40^ were also fewer in numbers, explaining the relatively narrow host range of *A. rabiei* due to lower adaptation. However, glutathione S-transferases (GSTs) were higher in abundance that functions in the detoxification of xenobiotic substrate, which may further aid in resistance against fungicides under field conditions. In addition, *A. rabiei* had a large inventory of CAZymes with a high capacity to degrade cellulose, pectin and xylan. These results correlated with the necrotrophic lifestyle of *A. rabiei*, where nutrition is obtained by degrading plant tissue.

Secretory proteins play crucial roles during early colonization and pathogenesis. Of 758 predicted secretory proteins, 546 were non-CAZymes and might be potential effector candidates. GO analysis showed that the majority of the secretory proteins are likely to respond to oxidative stress. These proteins may be secreted to counteract host-generated oxidative stress. The pathogenicity related proteins of *A. rabiei* effector reservoir included homologs of extracellular cutinase Pbc1 of *Pyrenopeziza brassicae*^41^, Glo1 and Gas1 of *U. maydis*^42^ and Atg15 of *M. oryzae*^43^. All these proteins play a major role in providing virulence to the pathogen. Other secreted proteins were lipases, hydrophobins and necrosis-inducing endopolygalacturonases in nature, which suggested that *A. rabiei* secretome consists of diverse proteins that function in an organised manner to suppress different aspects of plant immunity for causing disease successfully.

In summary, the present study has unlocked new prospects for the comprehensive genomic study of a variety of biological processes that make *A. rabiei* a successful necrotrophic pathogen. Detailed comparative genomics studies may provide unexpected new insights into biological phenomena of general interest. Functional characterization of potential effector candidates is a prerequisite for determining their roles in pathogenesis. Such studies will provide further insight and help in designing strategies to control this devastating disease and other necrotrophic fungal diseases.

## Methods

### Culture conditions, DNA isolation

The *A. rabiei* isolate ArD2 (Indian Type Culture Collection No. 4638) was obtained from the Division of Plant Pathology, Indian Agricultural Research Institute (New Delhi, India) and was used for whole genome sequencing. ArD2 is a highly virulent isolate with pinkish black spores. Vegetative mycelia were grown on potato dextrose agar (PDA; Difco Laboratories, USA) for 20 days or in potato dextrose broth (PDB; Difco Laboratories, USA) for 5 days at 22 °C in an incubator shaker at 120 rpm in the dark. Mycelial balls were harvested, and then total DNA was isolated using a DNeasy Plant Maxi kit (Qiagen) as per the manufacturer’s instructions.

### Genome sequencing and assembly

The genome of *A. rabiei* was sequenced using an Illumina HiSeq1000 sequencing platform. The DNA libraries of 200, 300, 500 and 200–500 bp inserts, along with a mate-pair library of 5 kb insert size, were generated for sequencing purposes. These libraries were then paired-end sequenced. The reads obtained from the Illumina sequencing were trimmed using FASTX-toolkit (v0.0.13.2) and bases having a quality score less than 20 were removed from both ends. After trimming, the reads with lengths <70 bp were discarded. The draft genome was assembled with the help of ABySS^5^ version 1.3.5 using the high-quality sequencing data using *k*-mer 23, and the gaps were filled using GapFiller^44^ version 1.11.

### Transposable elements and SSR identification

RepeatScout^11^ was used to identify *de novo* repetitive elements in the *A. rabiei* genome. It generated a library of 278 repetitive families with l-mer size 15, which included transposable elements (TEs) and dispersed duplicated sequences. This library was then filtered using following parameters: 1) Predicted repeats were aligned to genome assembly via BLASTN and hits were discarded if alignment length was <50 bp; 2) Repeats with frequency <5 in the genome were removed, and 3) Those repeats were also discarded for which significant hits to known proteins were found in Uniprot, except the ones showing hits to the known TEs. The resultant 155 consensus sequences were classified using TEclass^12^. Moreover, these repetitive families were also annotated using RepBase (http://www.girinst.org/repbase/index.html) by TBLASTX search. Then, the *A. rabiei* genome assembly was masked with 155 repetitive families using RepeatMasker^45^.

A high-throughput SSR search to identify mono- to hexanucleotide SSR motifs was performed using MIcroSAtellite identification tool (MISA) (http://pgrc.ipk-gatersleben.de/misa/download/misa.pl) with default parameters. The default parameters used were: minimum SSR motif length of 10 bp and repeat length of mono-10, di-6, tri-5, tetra-5, penta-5, and hexa-5; the maximum size of interruption allowed between two different SSRs in a compound sequence was 100 bp.

### Gene prediction

Protein-coding genes in the *A. rabiei* masked genome were predicted using three different gene prediction programs: GeneMark-ES^46^, Fgenesh^47^ and AUGUSTUS^48^. Fgenesh was trained with *S. nodorum* that predicted a total of 7,707 protein coding genes, while the unsupervised training program GeneMark-ES predicted 11,299 genes. For AUGUSTUS, *A. rabiei* ESTs were used as hints file and *S. nodorum*, *C. sativus* and *P. tritici-repentis* (all belongs to the order Pleosporales) were selected as default gene models. This resulted in prediction of 10,708, 11,293 and 10,843 protein coding genes, respectively. Altogether 51,850 genes predicted from all the three programs were used to retrain AUGUSTUS (with parameters from *C. sativus* as default gene model) and then new genes were predicted. Additionally, annotated proteins from *S. nodorum*, *C. sativus* and *P. tritici-repentis* were mapped onto the genome of *A. rabiei* using Exonerate: protein2genome. The resultant mapped genes from Exonerate were mapped back to the genes predicted by the retrained AUGUSTUS and only the genes which could be mapped were selected.

In order to evaluate the genome completeness, the highly conserved single or low copy genes were searched in the predicted proteins of *A. rabiei*. The BLASTP search was carried out against the single-copy families that contribute 246 single copy genes from all 21 species available in the FUNYBASE^49^. Additionally, 248 core eukaryotic genes (CEGs) were also searched by BLASTP. For both the approaches to assess the completeness, the cut-off E-values of ≤ 1e-5 was implemented.

### Genome annotation

For functional annotation of *A. rabiei* predicted genes, BLASTX search against NCBI non-redundant database was performed with cut-off E-values of ≤ 1e-5 and identity ≥40%. Gene ontology (GO) analysis was carried out using BLAST2GO^50^. For pathway analysis, the 10,596 protein sequences were annotated from the Kyoto Encyclopedia of Genes and Genomes (KEGG)^51^ using blastKOALA. A total of 3,423 predicted protein sequences were assigned KO identifiers. These assigned KO identifiers were used to map the KEGG database with help of KEGG mapper to identify the pathways. Pfam analysis was done by batch sequence search against Pfam database^52^ with E-value ≤ 1e-5 (http://pfam.xfam.org/). For CAZymes prediction, CAZymes Analysis Toolkit (CAT)^53^ was used. To identify the potential pathogenicity-related proteins, BLASTP search was performed against Pathogen-Host Interaction database (PHI-base)^26^ with threshold E- value of ≤ 1e-5. The tRNA genes were predicted using a combination of tRNAscan-SE^54^ and ARAGORN^55^. The nucleotide sequences of the assembled genome were used for prediction using default parameters and a eukaryotic gene model.

### Phylogenetic analysis

The phylogeny was performed using amino acid sequences of actin (ACT), beta-tubulin (BTUB), translation elongation factor-1 alpha (TEF1) and NAD-dependent glycerol-3-phosphate dehydrogenase (GPD). Protein sequences were downloaded from GenBank. The amino acid sequences were aligned in T-REX^56^ using MAFFT^57^ as the sequence alignment tool. ProtTest 3.2.1^58^ was used for the estimation of best-fit protein evolutionary model for ML analysis. The species tree was generated in T-REX using RAxML^59^ with LG model of evolution. The phylogenetic tree was visualized using FigTree (v1.4.) (http://tree.bio.ed.ac.uk/software/figtree/).

### Comparative analysis of orthologous gene families

The orthologous groups among *A. rabiei*, *S. nodorum*, *C. heterostrophus* and *P.tritici-repentis* were identified with the help of OrthoMCL^60^. Orthologous gene pairs were considered on the basis of the amino acid sequence similarity sharing upto 50% of the total length of the shorter gene being analyzed (BlastP, threshold E-value ≤ 1e-5).

### Secretome prediction and analysis

The 10,596 protein set of *A. rabiei* was analyzed in SignalP v4.1 for prediction of the secretory signal peptide. The protein sequences lacking the signal peptide (9,479) were analyzed by SecretomeP v1.0^27^ for the prediction of non-classical secretory proteins. Then the protein sequences approved from both the SignalP and SecretomeP were further analyzed by TargetP v1.1. After this, the protein sets were scrutinized for the presence of transmembrane domain using TMHMM v2.0 and, simultaneously, for the presence of GPI (glycosylphosphatidyl inositol)-anchor with big-PI FungalPredictor. Only the proteins having no transmembrane domain and one transmembrane domain within the N-terminal signal peptide were selected. Further, ProtComp v9.0 was employed to predict the localization of protein sequences obtained from both classical and non-classical pipeline, using the LocDB and PotLocDB databases Furthermore, the GPI-anchor proteins present in these extracellular predicted proteins were discarded. Finally, 538 proteins were predicted as classical secretory proteins and 220 proteins as non-classical secretory proteins resulting in a secretome of 758 protein sequences.

The predicted secretome was functionally annotated by assigning GO terms using BLAST2GO. The CAZymes Analysis Toolkit (CAT) was used to detect carbohydrate active enzymes (CAZymes) based on the CAZy database in the *A. rabiei* secretome. An annotation method “based on association rules between CAZy families and Pfam domains” was used with an E-value threshold of 0.01, a bitscore threshold of 55 and rule support level 40. The predicted secretory proteins that could not be annotated by any of the above approaches were analyzed for the presence of characteristic features of effector proteins. In such proteins, high cysteine residue content and tandem repeats were examined. The number of cysteine residues was identified using Perl script. Protein internal repeats were predicted using T-Reks (http://bioinfo.montp.cnrs.fr/?r=t-reks/). The *in planta* localization of mature effector proteins were predicted by WoLF PSORT (http://www.genscript.com/psort/wolf_psort.html). WoLF PSORT analysis was performed using “runWolfPsortSummaryplant”, which estimates localization sites with a sensitivity and specificity of approximately 70%. The NLS was predicted in the mature proteins using NLStradamus (http://www.moseslab.csb.utoronto.ca/NLStradamus/). The potential virulence-related proteins were identified by searching the predicted 758 secreted proteins of *A. rabiei* against the PHI-base with cut off E-values of ≤ 1e-5.

## Additional Information

**Accession numbers**. The Ascochyta rabiei whole genome shotgun project has been deposited at DDBJ/EMBL/GenBank under the accession JYNV00000000. The version described in this paper is the first version, JYNV01000000.

**How to cite this article**: Verma, S. *et al*. Draft genome sequencing and secretome analysis of fungal phytopathogen *Ascochyta rabiei* provides insight into the necrotrophic effector repertoire. *Sci. Rep*. **6**, 24638; doi: 10.1038/srep24638 (2016).

## Supplementary Material

Supplementary Information

Supplementary Dataset 1

Supplementary Dataset 2

Supplementary Dataset 3

Supplementary Dataset 4

Supplementary Dataset 5

Supplementary Dataset 6

Supplementary Dataset 7

Supplementary Dataset 8

Supplementary Dataset 9

Supplementary Dataset 10

Supplementary Dataset 11

Supplementary Dataset 12

Supplementary Dataset 13

## Figures and Tables

**Table 1:** Genome features of *A. rabiei*.

Features	*A. rabiei*
Size (Mb)	34.65
Coverage	178X
% (G + C) content	51.60
% Repeat	9.94
Protein-coding genes	10,596
Average gene length (bp)	1,726
Gene density (number of genes per Mb)	305
Average exons per gene	2.74
Average exon length (bp)	557
Average introns per gene	1.74
Average intron length (bp)	111
Single exon genes	2,859
tRNA genes	125
Secreted proteins	758
