# Supplementary material for: Draft genome sequencing and secretome analysis of fungal phytopathogen Ascochyta rabiei provides insight into the necrotrophic effector repertoire
Source: Sci Rep. 2016 Apr 19;6:24638. doi: 10.1038/srep24638 (PMC4835772; doi:10.1038/srep24638)
Supplement: Supplementary Information [file srep24638-s1.pdf]

Draft genome sequencing and secretome analysis of fungal phytopathogen *Ascochyta rabiei* provides insight into the necrotrophic effector repertoire

Sandhya Verma, Rajesh Kumar Gazara, Shadab Nizam, Sabiha Parween, Debasis Chattopadhyay & Praveen Kumar Verma*

National Institute of Plant Genome Research, Aruna Asaf Ali Marg, New Delhi-110067, India

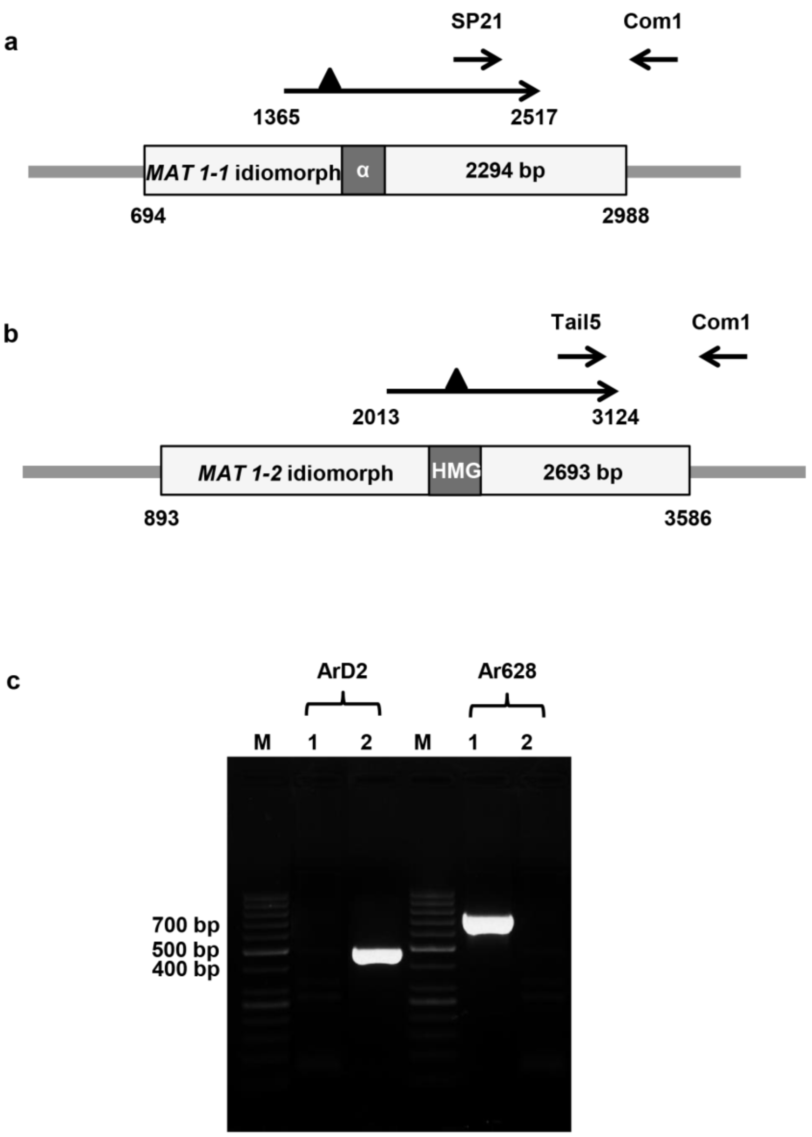

Supplementary Figure 1. Determination of the mating type of *A. rabiei* D2. The mating type was determined by PCR using *MAT* idiomorph-specific primers. **(a)** and **(b)** A schematic representation of the structural organization of *A. rabiei MAT1-1* and *MAT1-2*. **(c)** An ethidium bromide-stained 1% agarose gel showing bands obtained by PCR to detect the *MAT* locus. The lanes numbered 1 and 2 represent PCR with primer pairs Com1 (common flanking primer)/SP21 (*MAT1-1*-specific) and Com1/Tail 5 (*MAT1-2*-specific), respectively. The Ar628 strain of *A. rabiei* was used as a control for mating type 1.

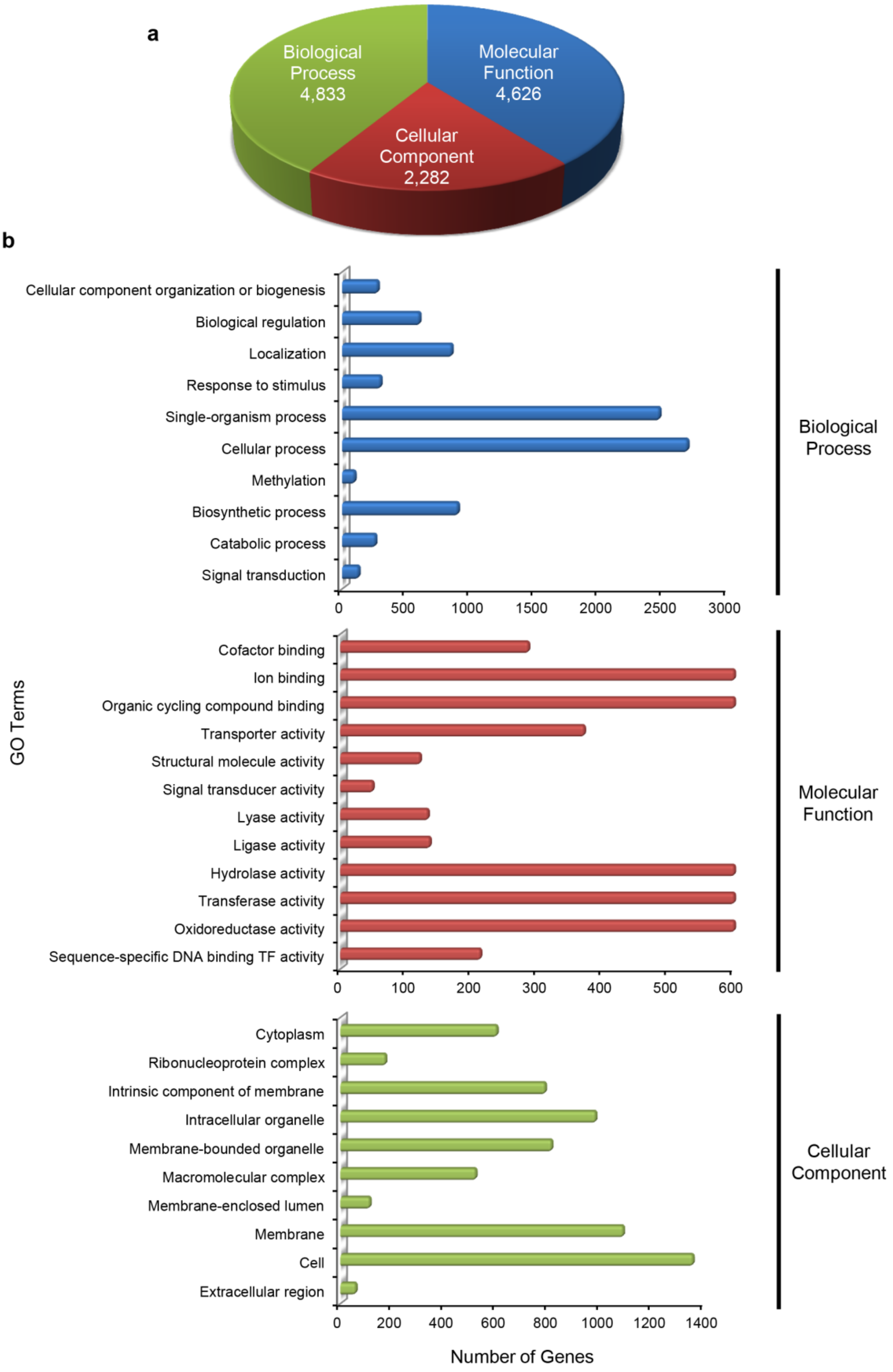
a
Biological Process 4,833
Molecular Function 4,626
Cellular Component 2,282
b
Cellular component organization or biogenesis
Biological regulation
Localization
Response to stimulus
Single-organism process
Cellular process
Methylation
Biosynthetic process
Catabolic process
Signal transduction
0 500 1000 1500 2000 2500 3000
Biological Process
Cofactor binding
Ion binding
Organic cycling compound binding
Transporter activity
Structural molecule activity
Signal transducer activity
Lyase activity
Ligase activity
Hydrolase activity
Transferase activity
Oxidoreductase activity
Sequence-specific DNA binding TF activity
0 100 200 300 400 500 600
Molecular Function
GO Terms
Cytoplasm
Ribonucleoprotein complex
Intrinsic component of membrane
Intracellular organelle
Membrane-bounded organelle
Macromolecular complex
Membrane-enclosed lumen
Membrane
Cell
Extracellular region
0 200 400 600 800 1000 1200 1400
Cellular Component
Number of Genes

Supplementary Figure 2. Gene Ontology (GO) functional annotations of predicted *A. rabiei* genes. GO classification was performed based on similarity searches against the NCBI non-redundant database using BLAST2GO software. **(a)** The genes were enriched into three primary GO categories. In total, 4,833 genes were assigned GO terms in the biological process category; however, 4,626 and 2,282 genes were assigned GO terms in the molecular function and cellular component categories, respectively. **(b)** Different terms under each GO category (biological process, molecular function and cellular component) are displayed. The major 10 categories in biological process, 12 categories in molecular function, and 10 categories in cellular component are shown. The *x*-axis represents the number of genes in a functional group, and the *y*-axis represents the GO terms.

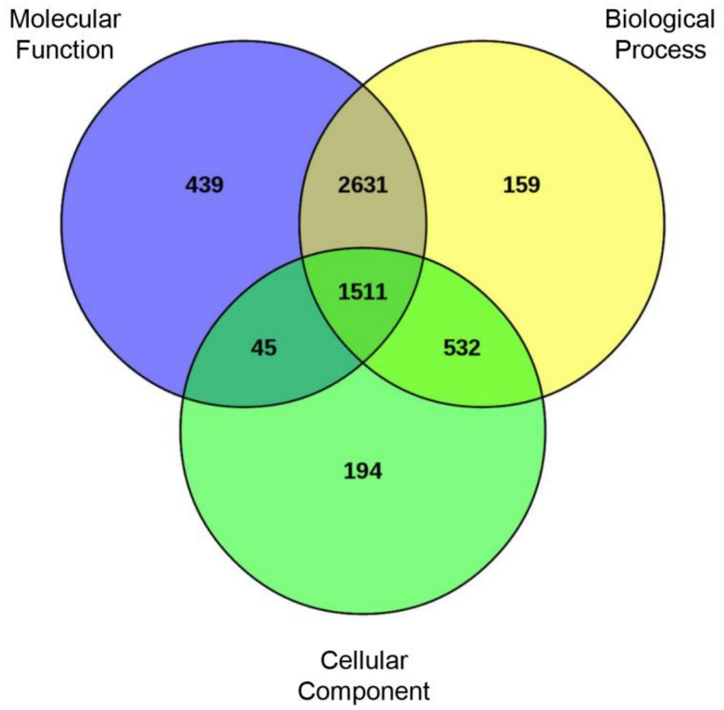

Supplementary Figure 3. The *A. rabiei* predicted genes assigned with Gene Ontology (GO) functional annotations. BLAST2GO software was used for GO classification based on similarity searches against the NCBI non-redundant database. In total, 5,511 genes (52% of total genes) were assigned GO terms in three primary categories. The Venn diagram shows *A. rabiei* genes having unique and shared GO terms. Almost 1,511 genes were assigned GO terms in all three categories, i.e., molecular function, biological process and cellular component. However, 439, 159 and 194 genes had GO terms unique to molecular function, biological process and cellular component, respectively.

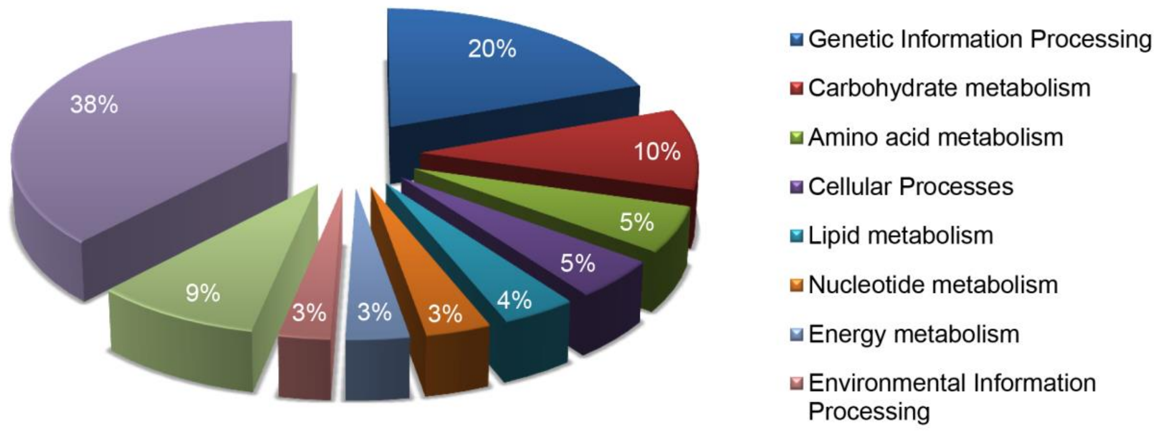

Supplementary Figure 4. Functional annotations of predicted *A. rabiei* proteins based on KEGG Orthology. Annotation was performed using the KEGG database and blastKOALA. The proteins were functionally categorized into 10 distinct categories.

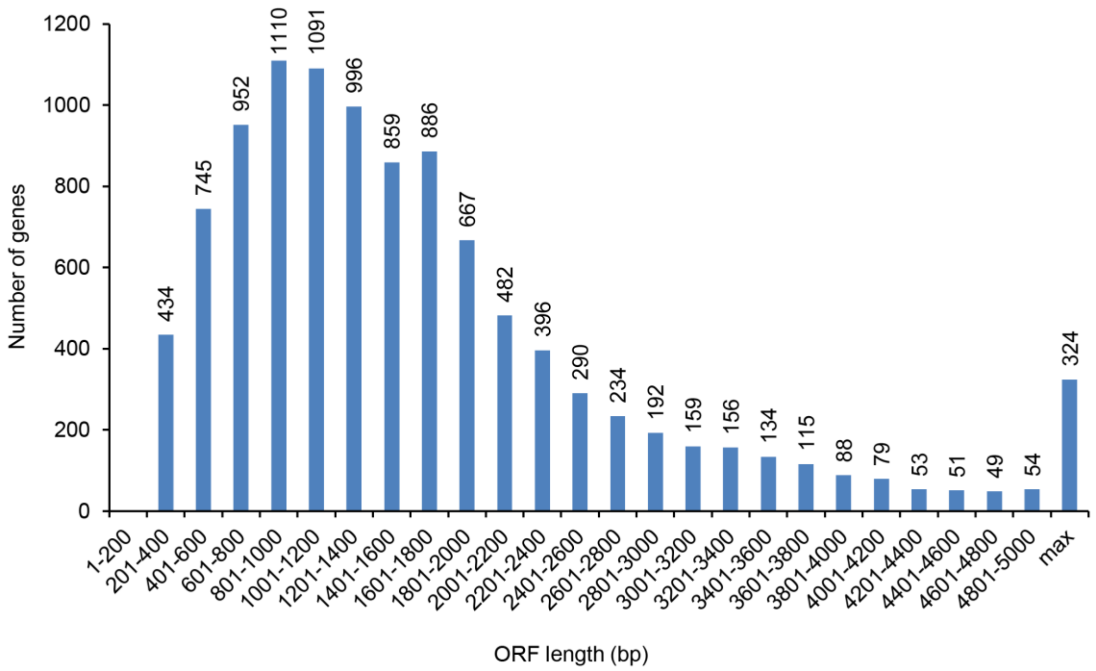

Supplementary Figure 5. ORF length distribution of the predicted genes of *A. rabiei*. The ORFs of lengths between 801-1000 bp were found to be the most abundant, followed by the ORFs of lengths between 1001-1200 bp.

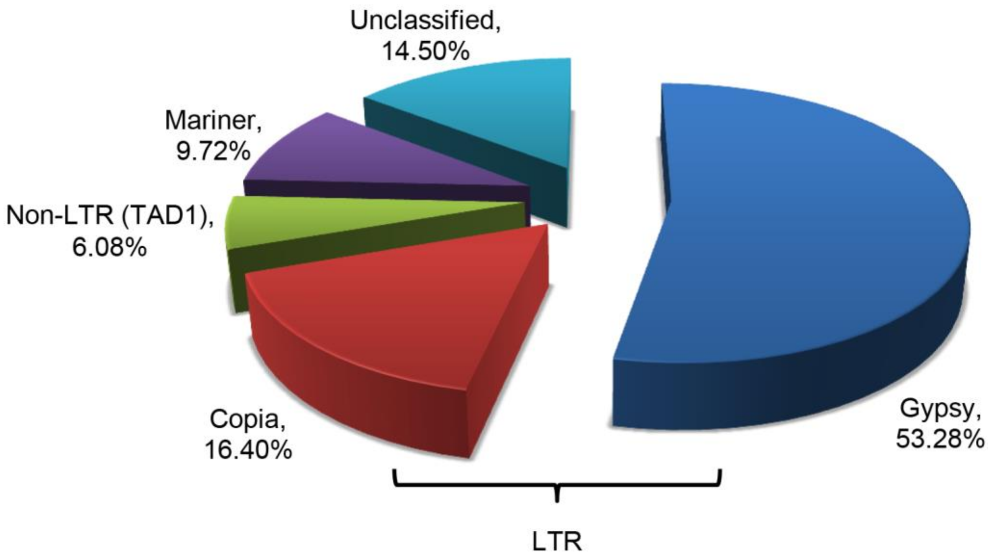

Supplementary Figure 6. Relative abundance of transposable elements in the *A. rabiei* genome. The proportion (%) of different types of repetitive sequences present in the *A. rabiei* genome. In the LTR type retrotransposons, *Gypsy* is the most abundant type of TE, followed by *Copia*. However, the non-LTR Tad1 is the least abundant form of TE. Repetitive elements were identified *de novo* using RepeatScout and then annotated manually by a TBLASTX search against RepBase.

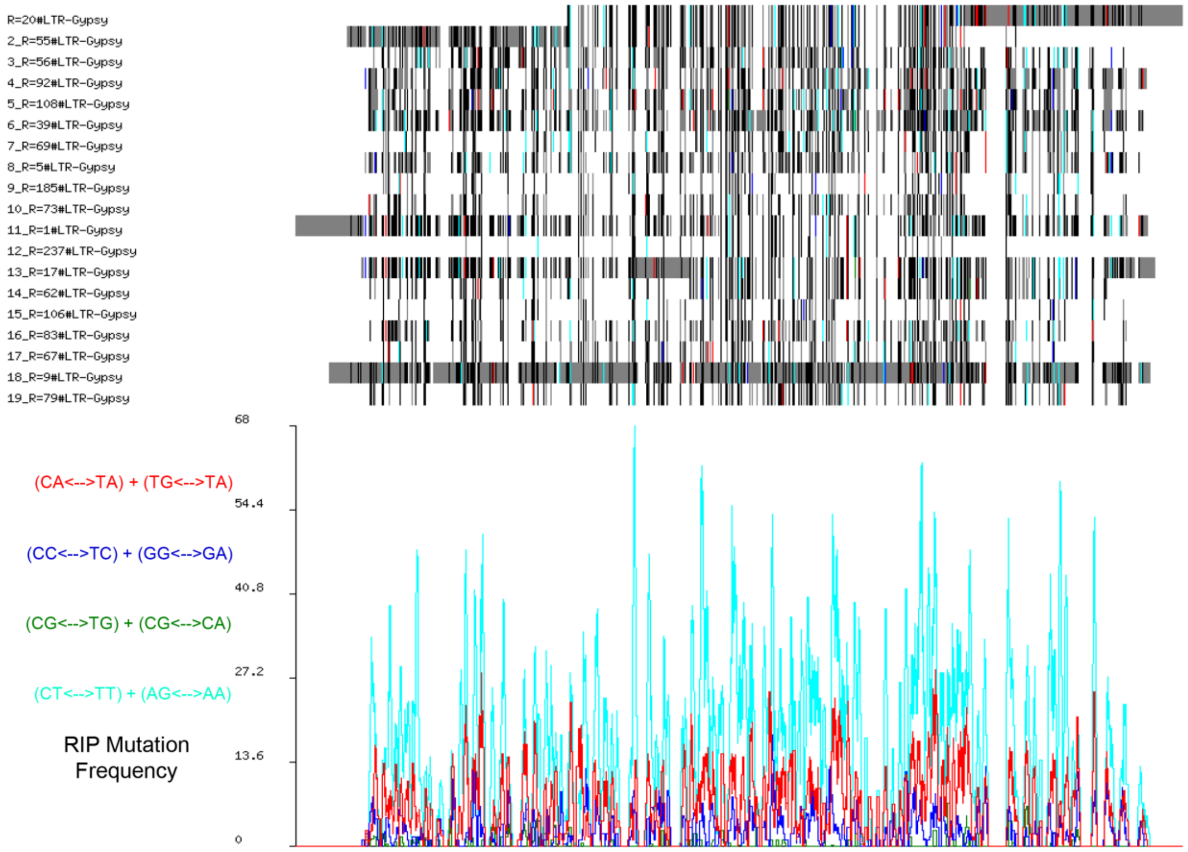

Supplementary Figure 7. RIP mutation in *Gypsy* class of LTR retrotransposons shown as RIPCAL output. The upper panel of output shows multiple alignment of the genomic regions corresponding to repeat units of each class. Polymorphic nucleotides are colored according to the type of RIP mutation detected. Black: invariant nucleotide; red: CpA↔TpA or TpG↔TpA mutations; dark blue: CpC↔TpC or GpG↔GpA mutations; green: CpG↔TpG or CpG↔CpA mutations; turquoise blue: CpT↔TpT or ApG↔ApA mutations. The lower panel of each output shows the frequency plot of the RIP mutations in accordance with the multiple alignment shown above over a rolling sequence window. Nucleotide polymorphisms are color-coded as in the upper panel.

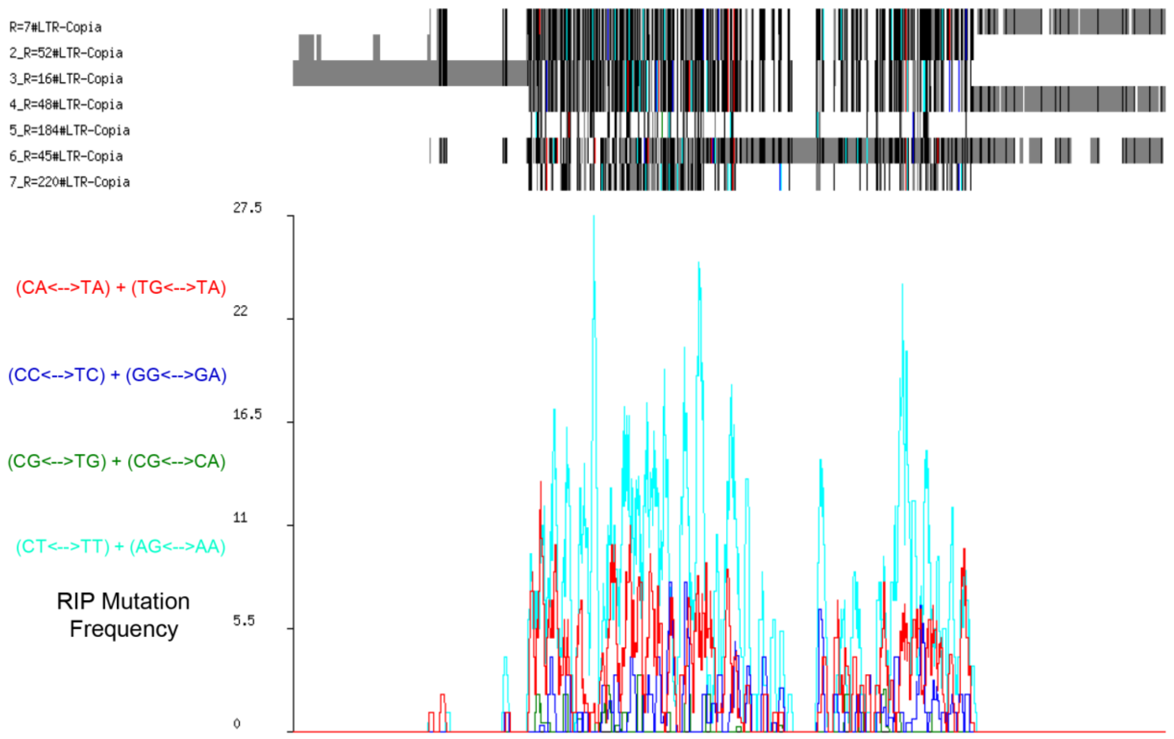

Supplementary Figure 8. RIP mutation in *Copia* class of LTR retrotransposons shown as RIPCAL output. The upper panel of output shows multiple alignment of the genomic regions corresponding to repeat units of each class. Polymorphic nucleotides are colored according to the type of RIP mutation detected. The lower panel of each output shows the frequency plot of the RIP mutations in accordance with the multiple alignment shown above over a rolling sequence window. Nucleotide polymorphisms are color-coded as in the upper panel.

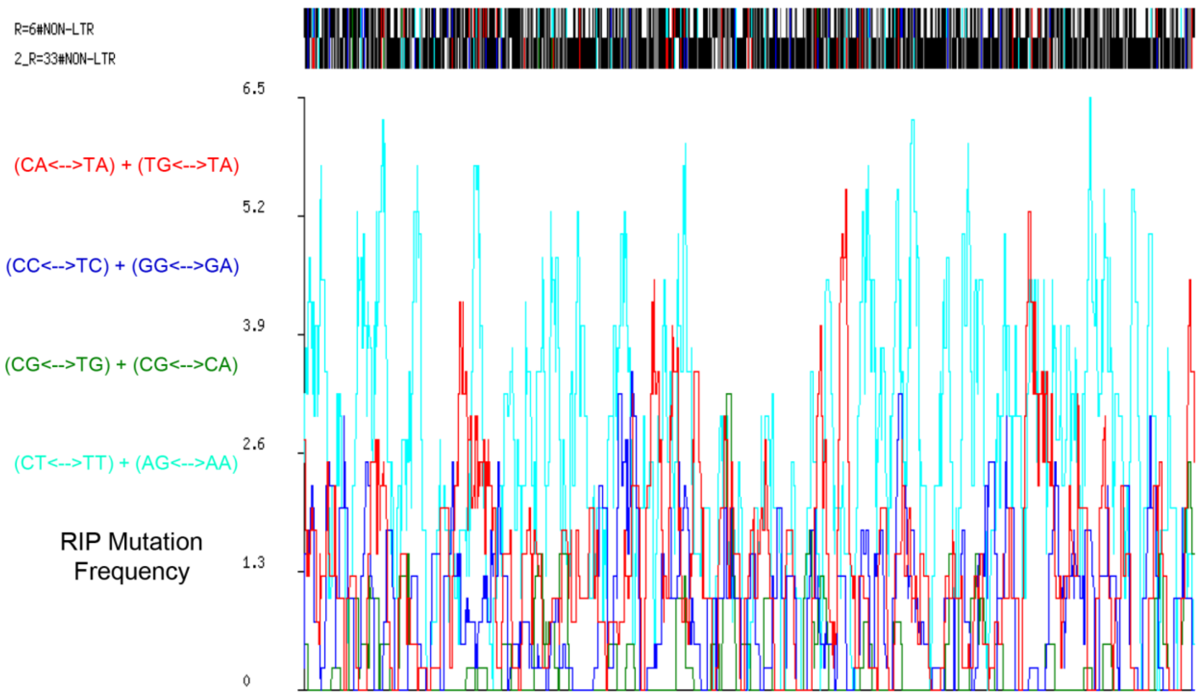

Supplementary Figure 9. RIP mutation in Tad1 class of non-LTR retrotransposons shown as RIPCAL output.

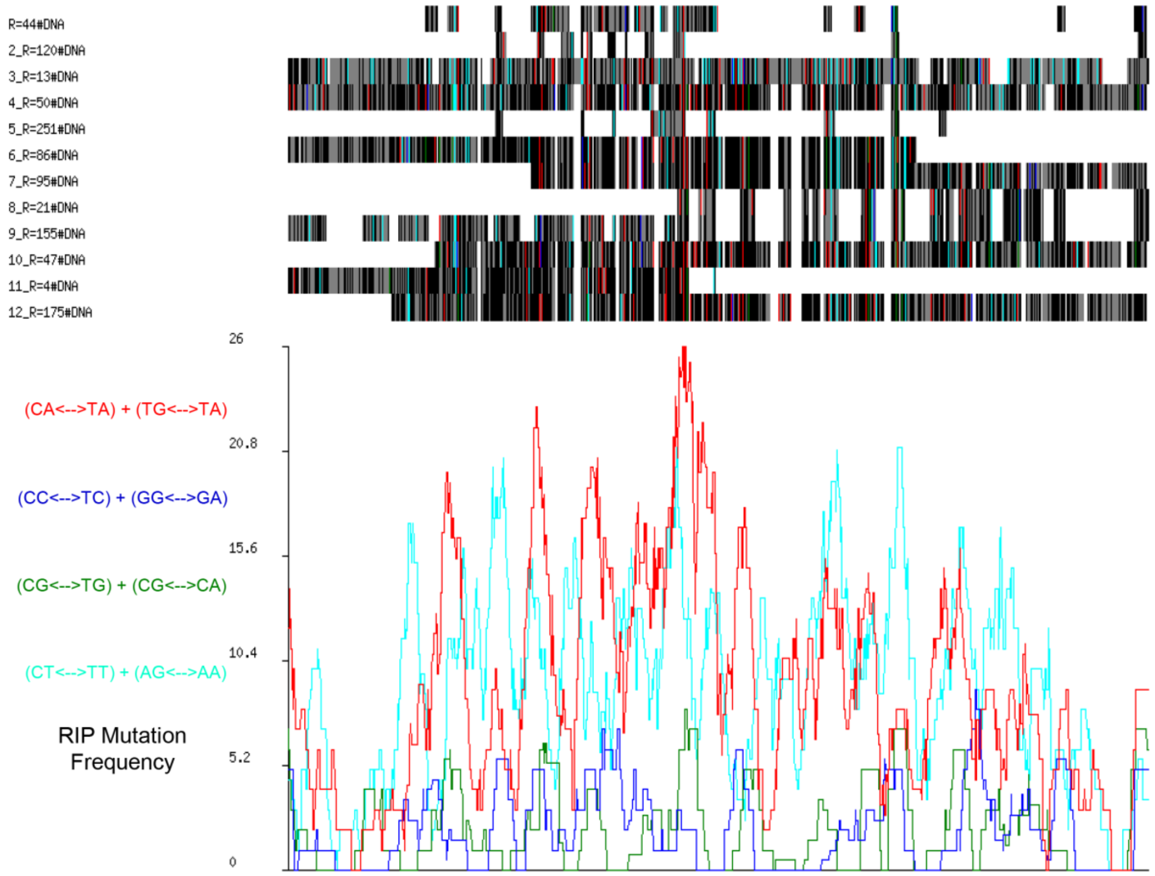

Supplementary Figure 10. RIP mutation in Tcl-Mariner class of DNA transposons shown as RIPCAL output.

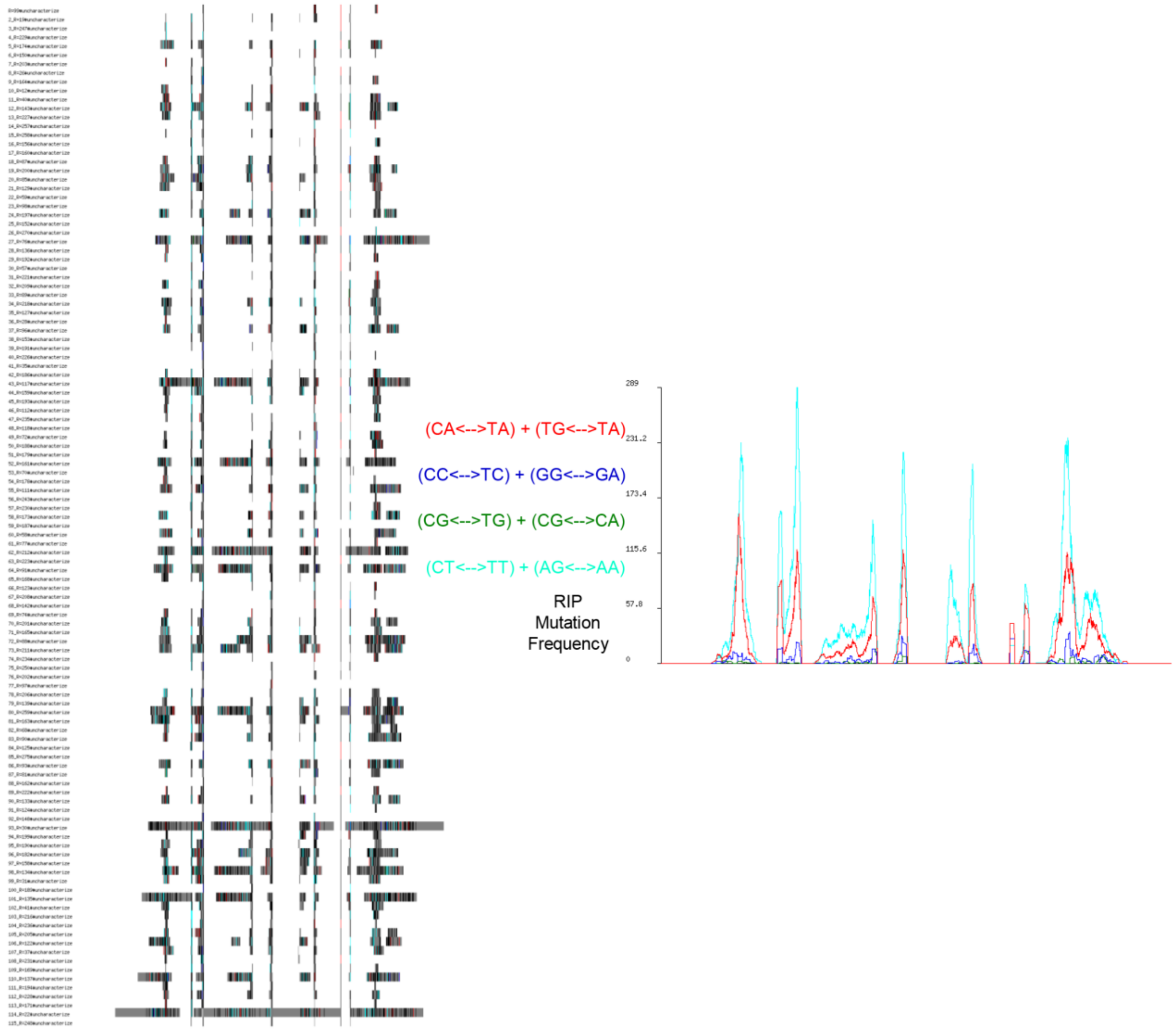

Supplementary Figure 11. RIP mutation in unclassified transposable elements shown as RIPCAL output. RIP mutation is shown for the unclassified repeat sequences.

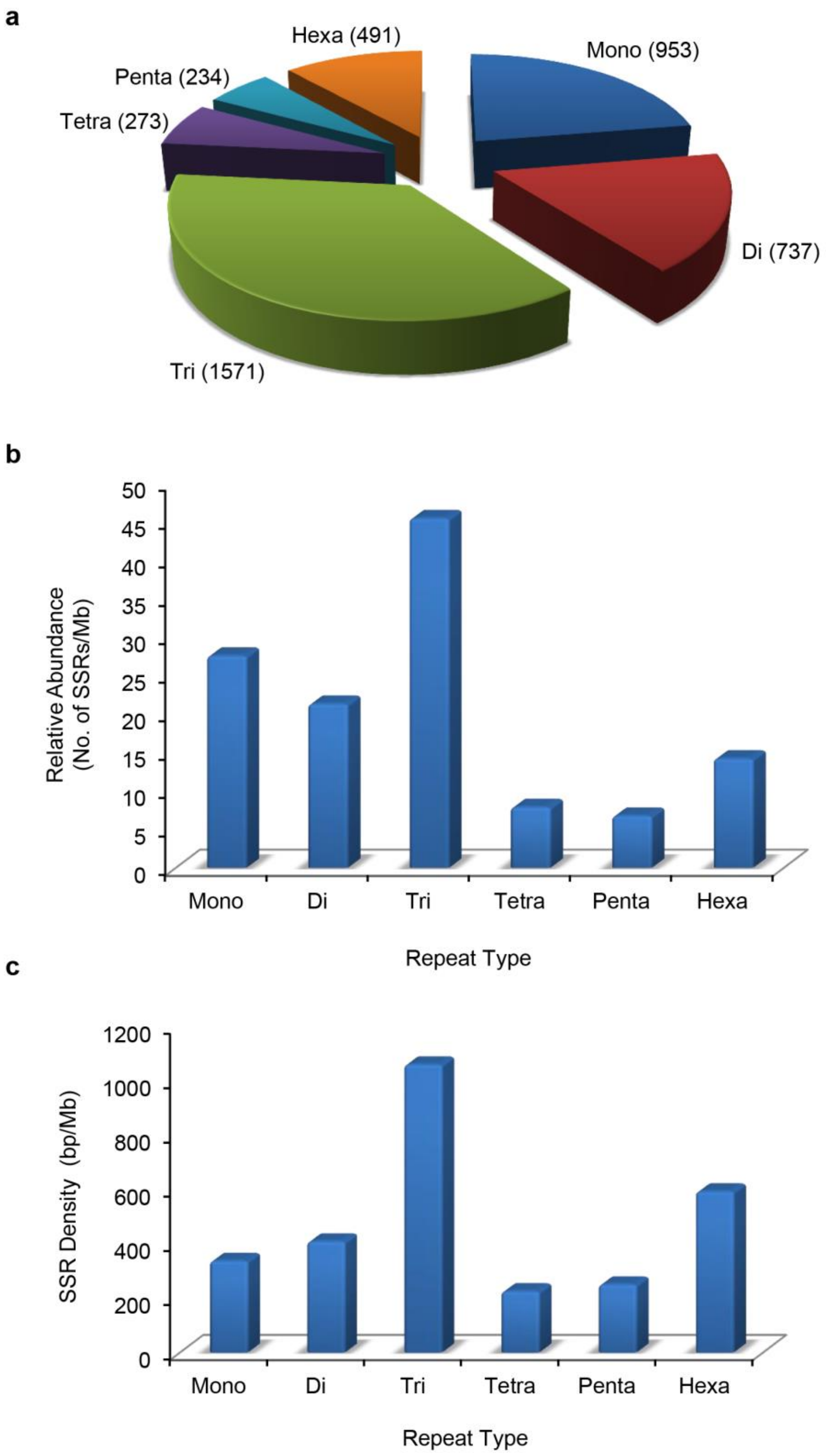

Supplementary Figure 12. SSRs across the genome of *A. rabiei*. (a) The total numbers of mono-, di-, tri-, tetra-, penta- and hexa-nucleotide SSRs present in the genome are shown.

Tri-nucleotide SSRs are the most dominant type of SSRs, whereas penta-nucleotide SSRs are least abundant. The total number of each SSR is shown in parenthesis. The SSRs were identified in the genome using MIcroSAtellite identification tool (MISA). **(b)** Relative abundance of SSRs across the *A. rabiei* genome is shown. Abundance is the total number of SSRs present per Mb of sequence analyzed. The tri-nucleotide repeat showed the highest relative abundance, while the penta-nucleotide repeat showed the lowest relative abundance. **(c)** Relative density of SSRs is shown. Density is the total sequence length (bp) contributed by each SSR per Mb of DNA of the analyzed sequence. The tri-nucleotide repeat showed the highest relative density, whereas the tetra-nucleotide repeat showed the lowest relative density.

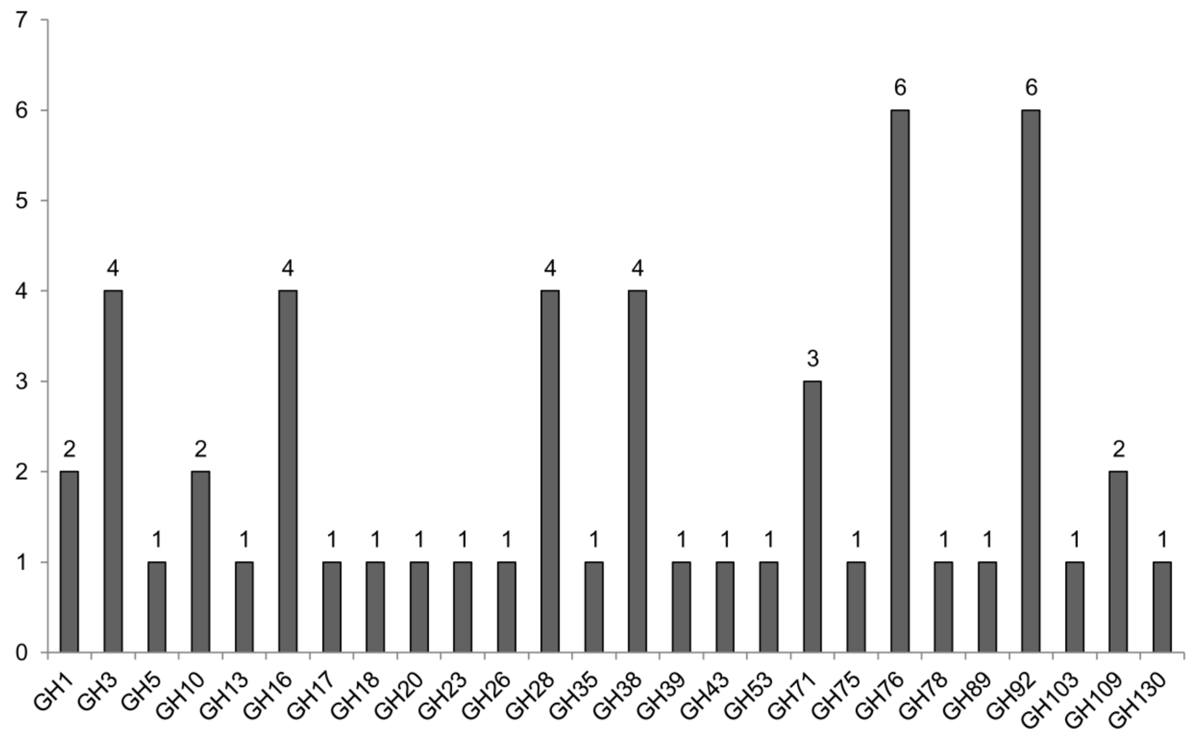

Supplementary Figure 13. The GHs present in gene families unique to *A. rabiei*. The bars represent the number of distinct GHs present in the 693 gene families that are unique to *A. rabiei* when compared to other closely related necrotrophic fungi i.e. *C. heterostrophus*, *P. tritici-repentis* and *S. nodorum*. The enzyme classes are indicated by codes that are defined by the CAZyme database.

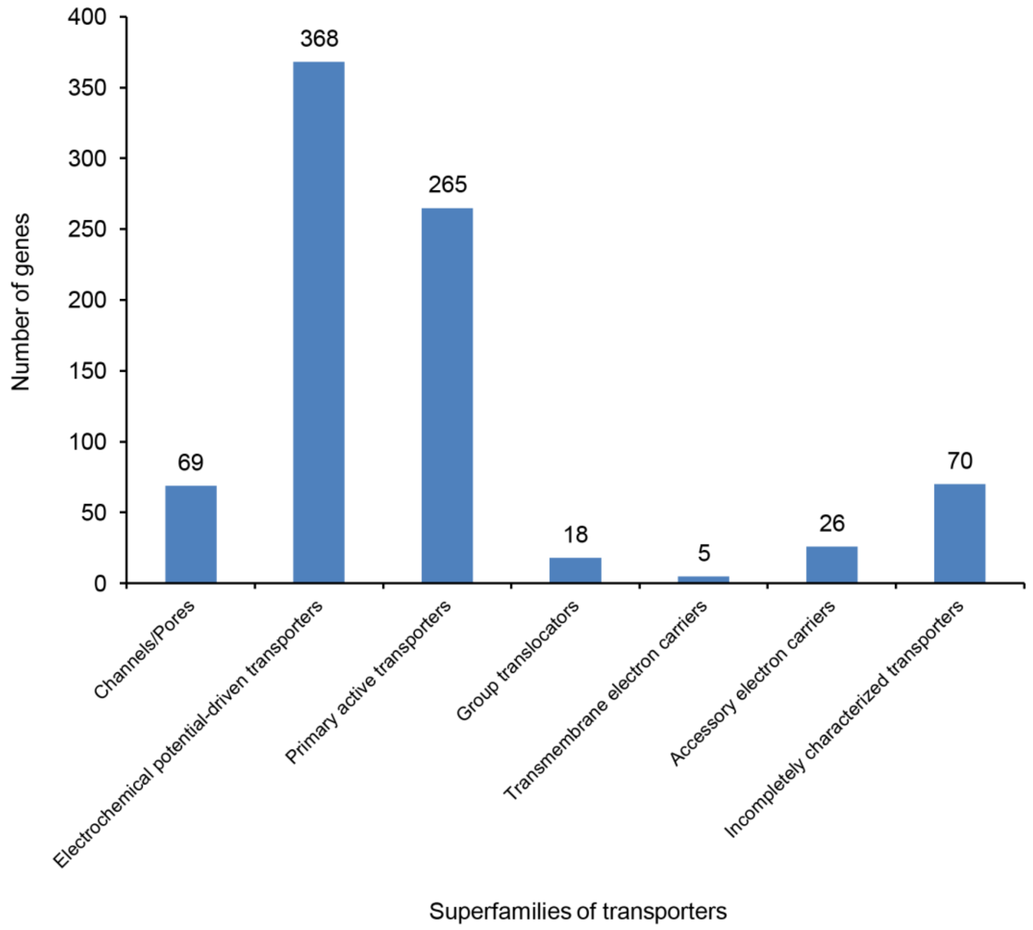

Supplementary Figure 14. Transporters identified in the *A. rabiei* genome. The identification and classification of transporters were performed based on similarity searches against the Transporter Classification Database (TCDB). The identified transporters were classified into seven distinct superfamilies, i.e., channels/pores, electrochemical potential-driven transporters, primary active transporters, group translocators, transmembrane electron carriers, accessory electron carriers and incompletely characterized transporters. The most abundant transporters belonged to the electrochemical potential-driven superfamily, followed by primary active transporters.

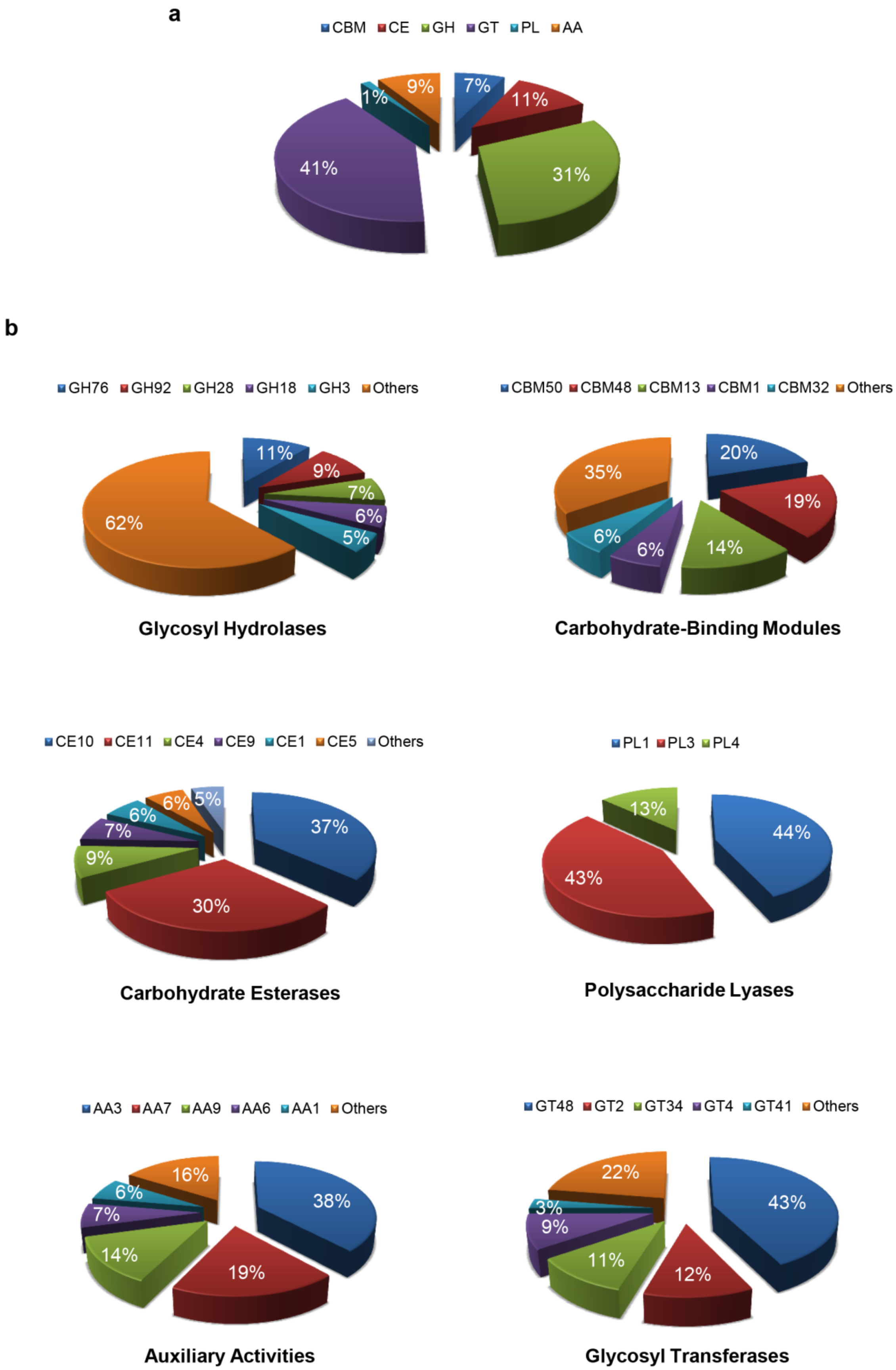
a
CBM CE GH GT PL AA
7%
11%
31%
41%
1%
9%
b
GH76 GH92 GH28 GH18 GH3 Others
11%
9%
7%
6%
5%
62%
Glycosyl Hydrolases
CBM50 CBM48 CBM13 CBM1 CBM32 Others
20%
19%
14%
6%
6%
35%
Carbohydrate-Binding Modules
CE10 CE11 CE4 CE9 CE1 CE5 Others
37%
30%
9%
7%
6%
6%
5%
Carbohydrate Esterases
PL1 PL3 PL4
44%
43%
13%
Polysaccharide Lyases
AA3 AA7 AA9 AA6 AA1 Others
38%
19%
14%
7%
6%
16%
Auxiliary Activities
GT48 GT2 GT34 GT4 GT41 Others
43%
12%
11%
9%
3%
22%
Glycosyl Transferases

Supplementary Figure 15. Carbohydrate-active enzyme (CAZyme) functional annotations of *A. rabiei* predicted genes. (a) Summary displayed for the six CAZyme categories: carbohydrate-binding modules (CBMs), carbohydrate esterases (CEs), glucoside hydrolases (GHs), glycosyl transferases (GTs), polysaccharide lyases (PLs) and auxiliary activities (AAs). **(b)** Distinct summaries of each of the CAZyme category representing the most abundant CAZyme classes. The prediction of CAZymes from the predicted genes of *A. rabiei* and their classification were performed using tools from the Carbohydrate-Active EnZymes (CAZyme) database.

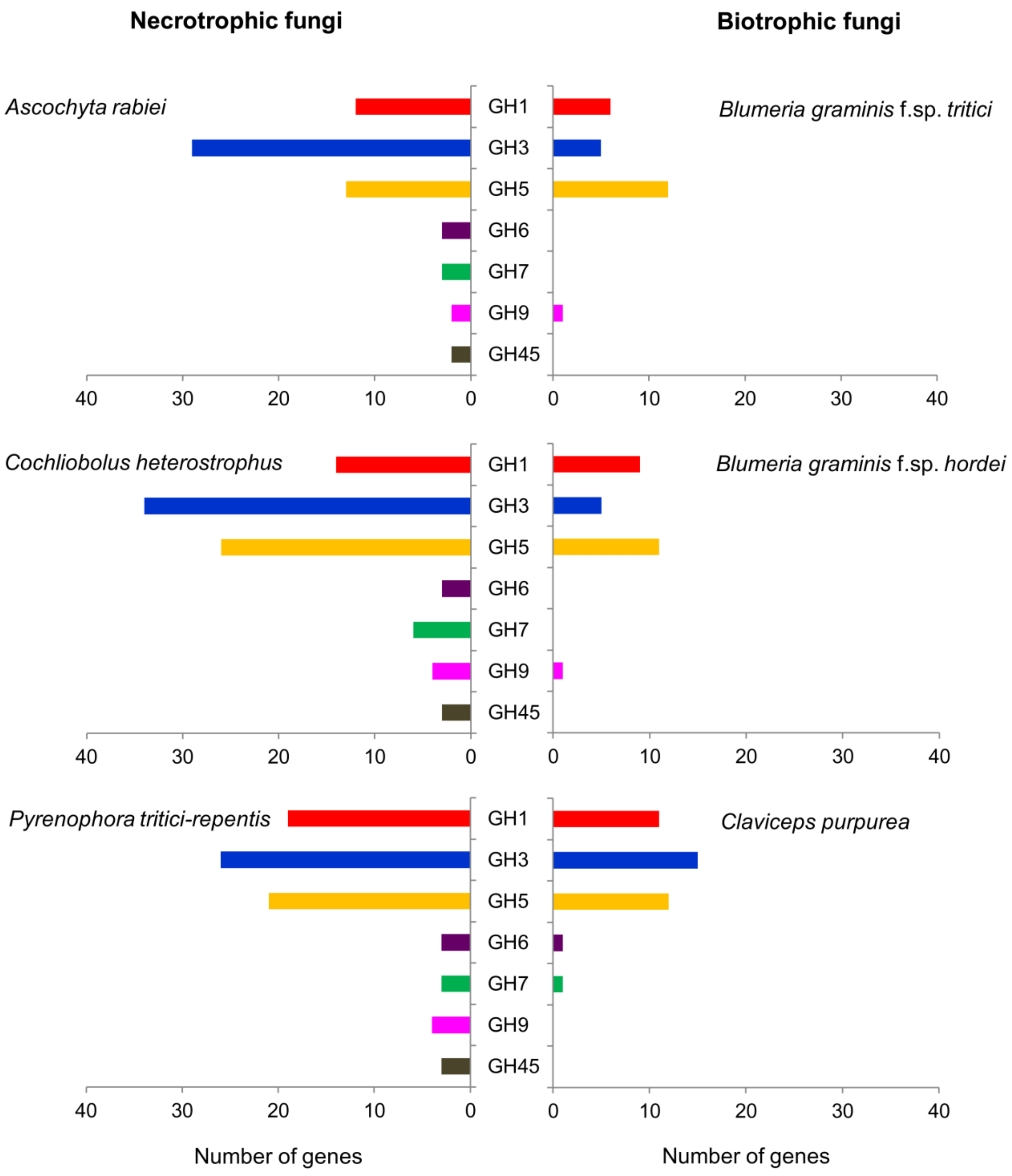

Supplementary Figure 16. Diversity of genes encoding CAZymes involved in cellulose degradation in *A. rabiei*, related necrotrophic fungi and biotrophic fungi. The bars are representing the number of cellulose degrading enzymes present in the genomes of six fungi.

On the left side, the necrotrophic fungi: *A. rabiei*, *C. heterostrophus* and *P. tritici-repentis* are shown. Whereas on right side, the biotrophic fungi: *Blumeria graminis* f.sp. *tritici*, *Blumeria graminis* f.sp. *hordei* and *Claviceps purpurea* are presented. The enzyme classes are indicated by codes that are defined by the CAZyme database.

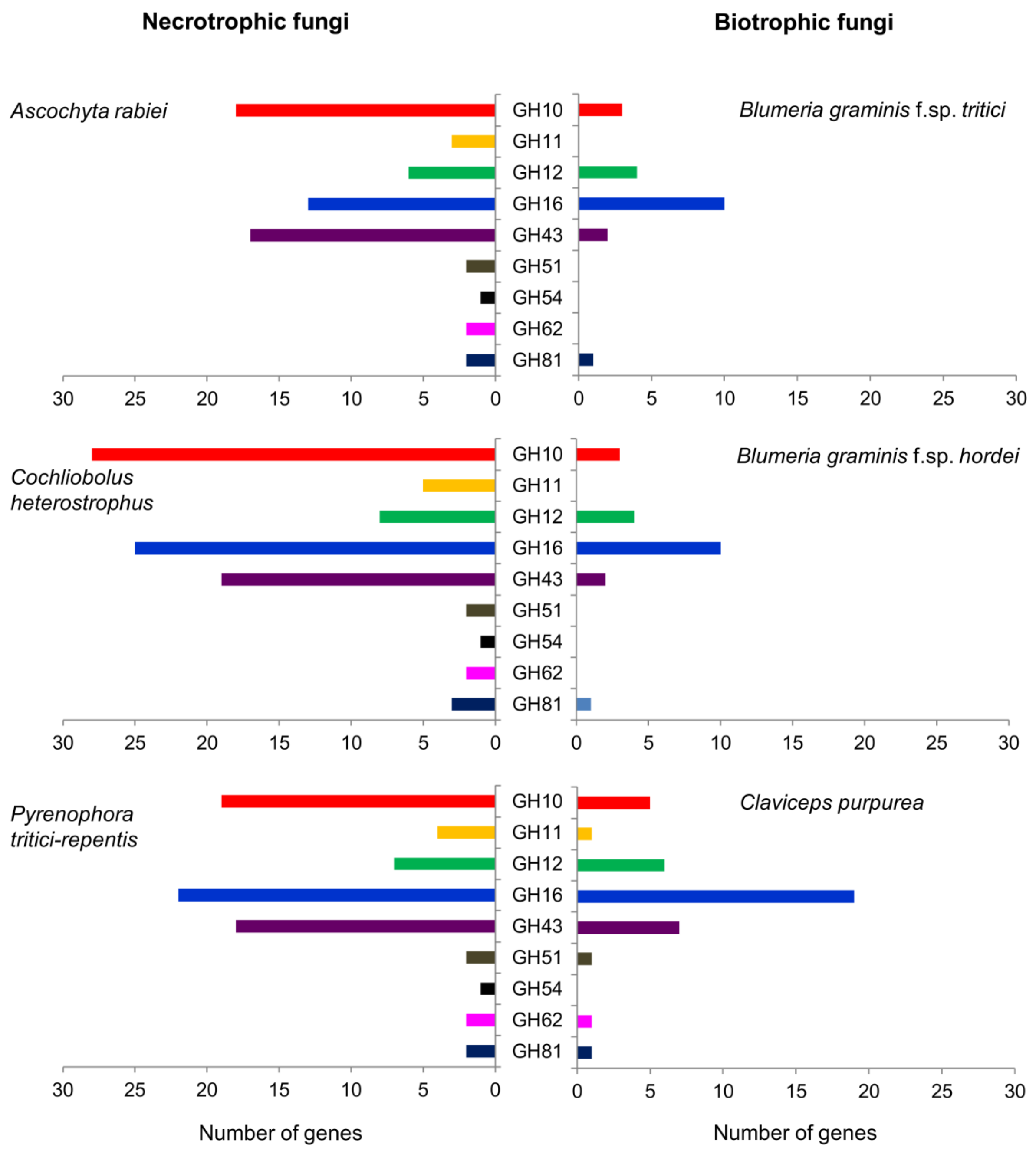

Supplementary Figure 17. Diversity of genes encoding CAZymes involved in hemicellulose degradation in *A. rabiei*, related necrotrophic fungi and biotrophic fungi. The bars are representing the number of hemicellulose degrading enzymes present in the genomes of six fungi. On the left side, the necrotrophic fungi: *A. rabiei*, *C. heterostrophus*

and *P. tritici-repentis* are shown. Whereas on right side, the biotrophic fungi: *Blumeria graminis* f.sp. *tritici*, *Blumeria graminis* f.sp. *hordei* and *Claviceps purpurea* are presented. The enzyme classes are indicated by codes that are defined by the CAZyme database.

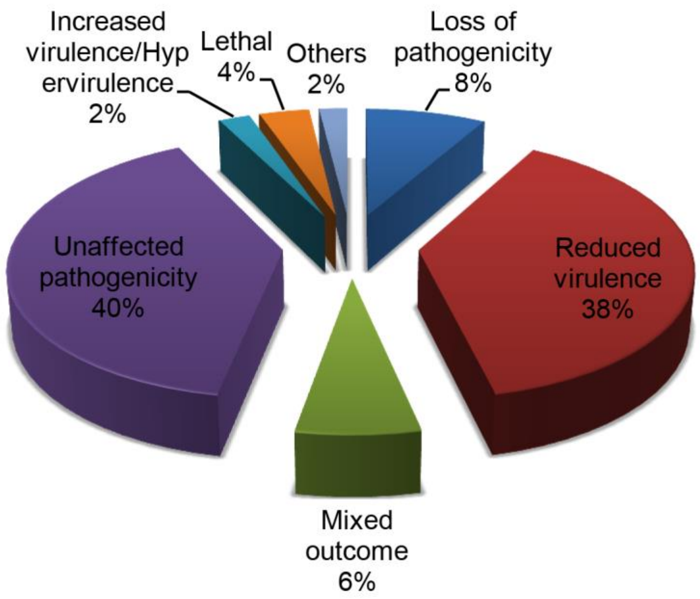

Supplementary Figure 18. Classification of *A. rabiei* genes based on the Pathogen-Host Interaction database (PHI-base). The orthologs of the PHI-base genes were predicted in *A. rabiei* using BLASTP search. Different mutant phenotypic categories of the PHI-base orthologs are shown. Almost 38% of the orthologs exhibited reduced virulence as their mutant phenotype, and 8% of mutants showed complete loss of pathogenicity.

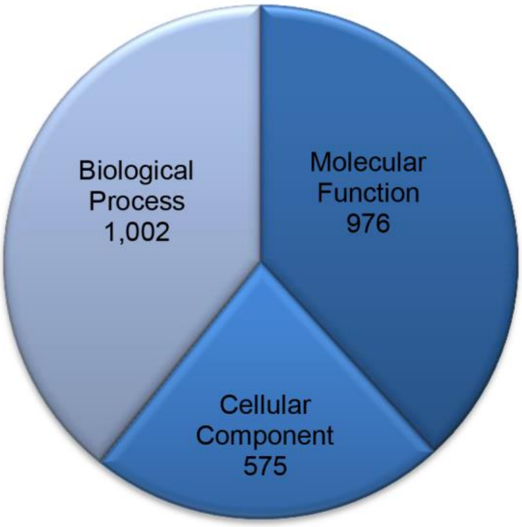

Supplementary Figure 19. Gene Ontology functional annotations of *A. rabiei* genes predicted to involve in pathogenicity by PHI-base. GO classification was performed for the genes that were predicted by PHI-base to function in pathogenicity. Classification was performed based on similarity searches against the NCBI non-redundant database using BLAST2GO software. In total, 1,002 genes were assigned GO terms in the biological process category; however, 976 and 575 genes were assigned GO terms in the molecular function and cellular component categories, respectively.

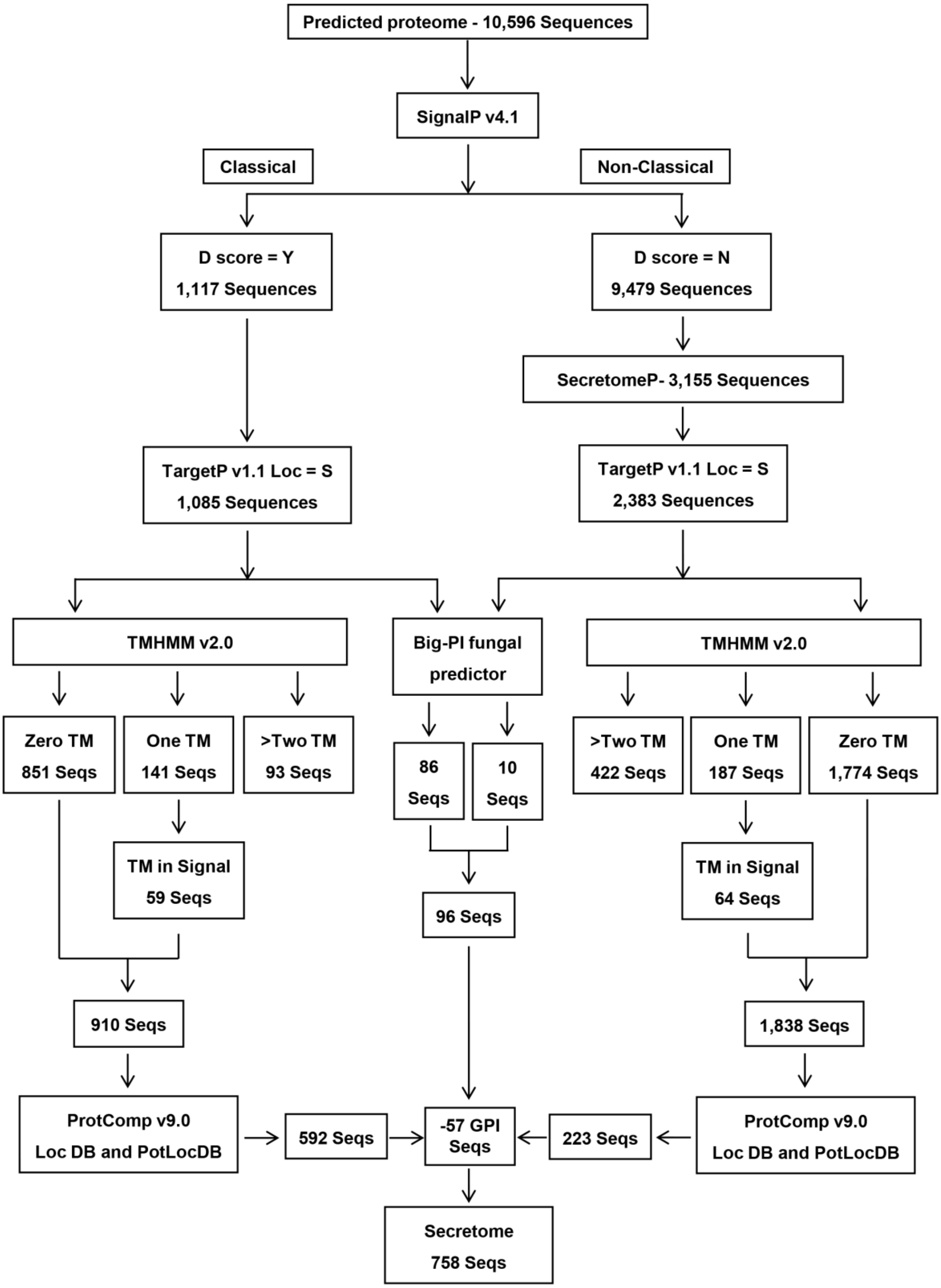

Predicted proteome - 10,596 Sequences
SignalP v4.1
Classical
Non-Classical
D score = Y
1,117 Sequences
D score = N
9,479 Sequences
SecretomeP- 3,155 Sequences
TargetP v1.1 Loc = S
1,085 Sequences
TargetP v1.1 Loc = S
2,383 Sequences
TMHMM v2.0
Big-PI fungal predictor
TMHMM v2.0
Zero TM
851 Seqs
One TM
141 Seqs
>Two TM
93 Seqs
86 Seqs
10 Seqs
>Two TM
422 Seqs
One TM
187 Seqs
Zero TM
1,774 Seqs
TM in Signal
59 Seqs
96 Seqs
TM in Signal
64 Seqs
910 Seqs
1,838 Seqs
ProtComp v9.0
Loc DB and PotLocDB
592 Seqs
-57 GPI Seqs
223 Seqs
ProtComp v9.0
Loc DB and PotLocDB
Secretome
758 Seqs

Figure 20. Overview of the computational pipeline used to identify the secretome of *A. rabiei*. Two approaches were used simultaneously to predict both the classical and non-classical secreted effector proteins of *A. rabiei*. In total, 758 proteins were predicted in the secretome of *A. rabiei*.

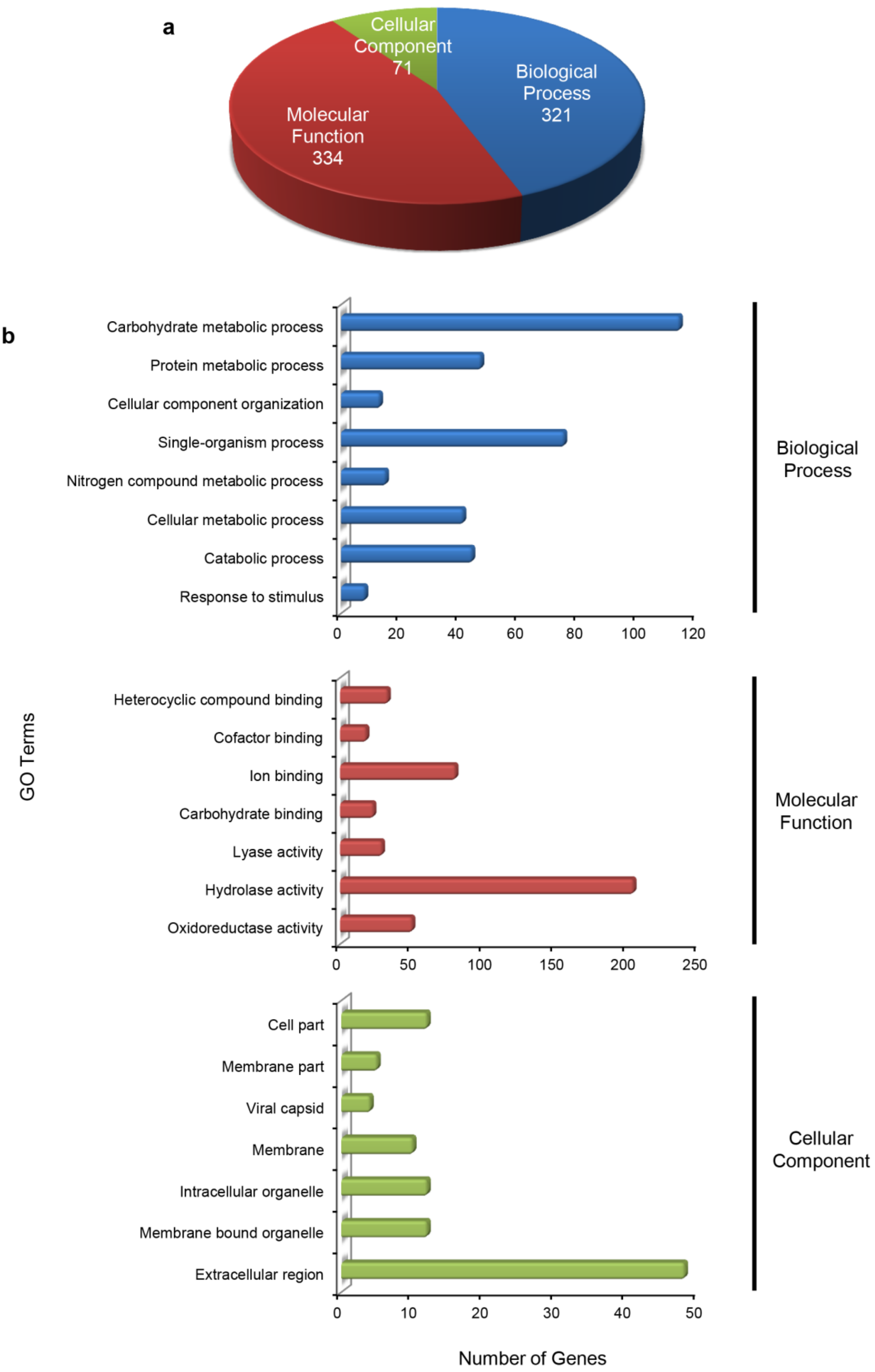
a
Cellular Component 71
Biological Process 321
Molecular Function 334
b
Carbohydrate metabolic process
Protein metabolic process
Cellular component organization
Single-organism process
Nitrogen compound metabolic process
Cellular metabolic process
Catabolic process
Response to stimulus
0 20 40 60 80 100 120
Biological Process
Heterocyclic compound binding
Cofactor binding
Ion binding
Carbohydrate binding
Lyase activity
Hydrolase activity
Oxidoreductase activity
0 50 100 150 200 250
Molecular Function
Cell part
Membrane part
Viral capsid
Membrane
Intracellular organelle
Membrane bound organelle
Extracellular region
0 10 20 30 40 50
Cellular Component
GO Terms
Number of Genes

Supplementary Figure 21. Gene Ontology functional annotation of the *A. rabiei* predicted secretome. GO terms were assigned to the protein-coding genes of the *A. rabiei* secretome based on similarity searches against the NCBI non-redundant database using BLAST2GO software. **(a)** Based on significant GO terms, the genes were enriched into three primary GO categories: biological process, molecular function and cellular component. In total, 334 genes were assigned GO terms in the molecular function category; however, 321 and 71 genes were assigned GO terms in the biological process and cellular component categories, respectively. **(b)** Different terms under each GO category are displayed. The major 8 categories in biological process and 7 categories each in molecular function and cellular component are shown. The *x*-axis represents the number of genes in a functional group, and *y*-axis represents the GO terms.

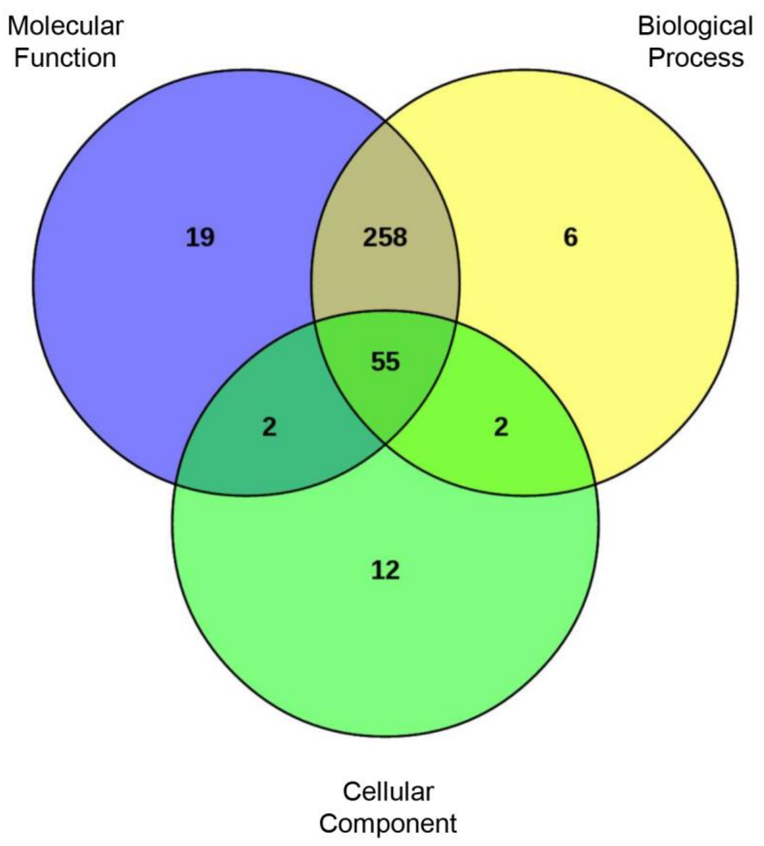

Supplementary Figure 22. Gene Ontology functional annotation of the protein-coding genes of the *A. rabiei* secretome. The GO classification was performed based on similarity searches against the NCBI non-redundant database using BLAST2GO software. In total, 354 genes (46.7% of secretome) were assigned GO terms in three primary categories. The Venn diagram shows protein-coding genes of the *A. rabiei* secretome with unique and shared GO terms. Approximately 55 genes were assigned GO terms in all three categories, i.e., molecular function, biological process and cellular component. However, only 19, 6 and 12 genes had GO terms unique to molecular function, biological process and cellular component, respectively. In total, 258 genes shared GO terms in molecular function and biological process categories.

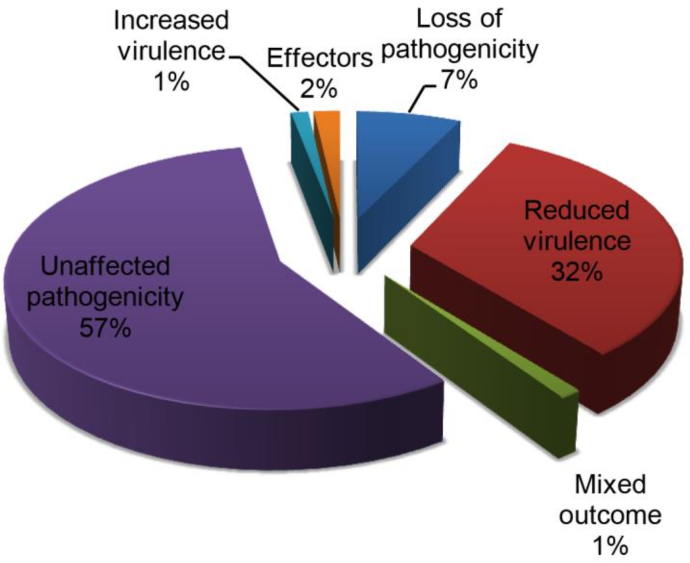

Supplementary Figure 23. Classification of the *A. rabiei* secretome based on the Pathogen-Host Interaction database (PHI-base). The orthologs of the PHI-base genes were predicted in the *A. rabiei* secretome using BLASTP search. Different mutant phenotypic categories of the PHI-base orthologs are shown. Approximately 57% of the identified orthologs showed mutant phenotypes of unaffected pathogenicity. However, almost 32% of the orthologs exhibited reduced virulence as their mutant phenotype, and 7% mutants showed complete loss of pathogenicity.

Supplementary Table 1. Description of libraries prepared for *A. rabiei* genome sequencing.

	**Library Type**	**Size**
Phase I	Paired-end library	200 bp
	Paired-end library	300 bp
	Paired-end library	500 bp
Phase II	Paired-end library	200-500 bp

Supplementary Table 2. Description of *A. rabiei* genome assembly.

**Features**	***A. rabiei***
Scaffold count	338
Scaffold total length (bp)	34,658,250
Scaffold minimum length (bp)	20,024
Scaffold maximum length (bp)	1,160,210
Scaffold average length (bp)	102,539.2
Scaffold N50 (bp)	154,808
Scaffold N90 (bp)	42,962

Supplementary Table 3. The *A. rabiei* predicted genes indicating the hits obtained from various public databases.

**Total genes**	**10,596**
Nr	9,248 (87.27%)
GO	5,511 (52.01%)
KEGG	3,424 (32.31%)
Pfam	7,118 (67.18%)
CAZymes	1,727 (16.30%)
PHI	2,707 (25.55%)
**Total**	**9,452 (89.20%)**

The numbers in the parentheses shows the percentages of the genes having significant hits in the respective databases (E-value < 1e-5).

Supplementary Table 4. The tRNA genes identified in *A. rabiei* along with their respective anticodons.

**Amino Acid**	**Anticodons**					
Alanine: 7	AGC: 6	GGC: 0	CGC: 0	TGC: 1		
Arginine: 6	ACG: 0	GCG: 0	CCG: 1	TCG: 2	CCT: 2	TCT: 1
Asparagine: 4	ATT: 0	GTT: 4				
Aspartate: 6	ATC: 0	GTC: 6				
Cysteine: 1	ACA: 1	GCA: 0				
Glutamate: 9			CTC: 6	TTC: 3		
Glutamine: 1			CTG: 1	TTG: 0		
Glycine: 13	ACC: 0	GCC: 9	CCC: 2	TCC: 2		
Histidine: 1	ATG: 0	GTG: 1				
Isoleucine: 7	AAT: 6	GAT: 0		TAT: 1		
Leucine: 8	AAG: 5	GAG: 0	CAG: 2	TAG: 0	CAA: 0	TAA: 1
Lysine: 8			CTT: 6	TTT: 2		
Methionine: 6			CAT: 6			
Phenylalanine: 4	AAA: 0	GAA: 4				
Proline: 1	AGG: 0	GGG: 0	CGG: 0	TGG: 1		
Serine: 11	AGA: 4	GGA: 0	CGA: 2	TGA: 2	ACT: 0	GCT: 3
Threonine: 10	AGT: 5	GGT: 0	CGT: 3	TGT: 2		
Tryptophan: 3			CCA: 3			
Tyrosine: 4	ATA: 0	GTA: 4				
Valine: 8	AAC: 6	GAC: 0	CAC: 1	TAC: 1		
Selenocysteine: 2				TCA: 2		
Supressor tRNAs: 2			CTA: 1	TTA: 1		
Unknown istoypes: 2						
Pseudo-tRNAs: 1						
Total tRNAs: 125						

tRNAs with introns: 94

Transfer RNAs were predicted using tRNAscan-SE with standard settings.

Supplementary Table 5. Classification of transposable elements by TEclass.

**Category**	**Numbers**
DNA Transposons	38
LTRs	72
LINEs	1
SINEs	6
Unclassified	38

Supplementary Table 6. Summary of transposable elements of *A. rabiei*.

**TEs Type**	**TEs Annotation**	**Number of Families**	**Number of Elements**	**Length Occupied (bp)**	**% of Genome Coverage**
**Long Terminal Repeats (LTR) Retrotransposons**	Gypsy	19	1,205	1,836,011	5.29%
	Copia	7	464	565,013	1.93%
**Non Long Terminal Repeats (non-LTR) Retrotransposons**	Tad1	2	194	209,657	0.60%
**DNA Transposons**	Tcl-Mariner	12	800	335,061	0.97%
**Unclassified**		115	1,814	499,597	1.63%
**Total**		**155**	**4,477**	**3,445,339**	**10.42%**

Supplementary Table 7. Essential components of gene silencing machinery identified in the genome of *A. rabiei* orthologous to *Neurospora crassa* [97].

**Process/ Components**	**Function**	***A. rabiei*** [a]	***N. crassa*** [b]	**E-value**	**Identity (%)**
RIP					
RID (RIP Defective)	Putative methyltransferase, necessary for RIP	ST47_g9657	NCU02034.7	2e-54	28.67
Dim-5	Histone H3-K9 methyltransferase, necessary for RIP	ST47_g6113	NCU04402.7	7e-60	38.49
RNA silencing Quelling					
QDE-1	RNA-dependent RNA polymerase (RdRP), necessary for quelling	ST47_g4168	NCU07534.7	7e-91	29.28
QDE-2	Argonaute-like, necessary for quelling	ST47_g3147	NCU04730.7	4e-128	31.49
QDE-3	RecQ family helicase, necessary for quelling	ST47_g3770	NCU08598.7	0	43.73
DCL1	Dicer-like protein 1, ATP-dependent helicase, involved in quelling	ST47_g4895	NCU08270.7	0	41.1
DCL2	Dicer-like protein 2, ATP-dependent helicase, involved in quelling	ST47_g10058	NCU06766.7	5e-132	37.14
QIP	QDE-2-interacting protein, involved in	ST47_g3703	NCU00076.7	6e-63	31.08

	quelling				
MSUD	RNA dependent RNA polymerase (RdRP), necessary for MSUD				
SAD-1		ST47_g9006	NCU02178.7	0	36
DNA methylation					
Dim-2	DNA methyltransferase	ST47_g2710	NCU02247.7	5e-110	35.38
HP1	Heterochromatin Protein 1, necessary for CpN methylation	ST47_g585	NCU04017.7	2e-29	41.49

[a] GenBank accession number of *N. crassa* genes.

[b] ID of orthologous of *N. crassa* genes in *A. rabiei*.

Abbreviations: RIP, Repeat Induced Point mutation; QDE, Quelling-Defective; MSUD, Meiotic Silencing of Unpaired DNA; SAD, Suppressor of Ascus-Dominance.

Supplementary Table 8. MISA results in the genome survey.

**Category**	**Numbers**
Total number of scaffolds examined	338
Total size of examined sequences (bp)	34,658,250
Total number of identified SSRs	4,259
Number of SSR containing scaffolds	307
Number of scaffolds containing more than 1 SSR	267
Number of SSRs present in compound formation	615
Total sequence length of SSRs (bp)	99,476
**Relative abundance** (No. of SSRs/Mb)	**123.09**
**Relative density** (Sequence length of SSRs (bp)/Mb)	**2,875.02**

Supplementary Table 9. Number of SSRs and length occupied by them in the genome.

Repeat Type	Number of SSRs	Length occupied (bp)
Mononucleotide	953	11,673
Dinucleotide	737	14,148
Trinucleotide	1571	36,684
Tetranucleotide	273	7,792
Pentanucleotide	234	8,665
Hexanucleotide	491	20,514

Supplementary Table 10. Number of compound SSRs in the genome.

Compound SSRs	Number
Total number of compound SSRs	448
Interrupted SSRs (C)	432 (96.43%)
Uninterrupted SSRs (C*)	16 (3.57%)

Supplementary Table 11. Most frequent SSR motifs in the genome. The repeat frequency are shown in closed brackets.

Repeat Unit				
**Di**	**Tri**	**Tetra**	**Penta**	**Hexa**
AG (126)	CAC (62)	ATCA (11)	TGTGC (6)	CCAGCA (9)
TC (79)	CAG (58)	AGGA/ATTA/ GATT (8)	CAAAG/CAGCA (4)	AAGAGA/CAGCAA/ GCGTGG (4)
GA (75)	AAG/TGC (55)	TGGA (7)	ACACC/AGGAA/ CACAG (3)	ACCAGC/CAACAG/ CATCAC (3)
TG (74)	TCG (52)	CCAT/GATG/ GTGA (6)	AAATC/AACCC/ ACAGG (2)	ACCTCG/ACGCCC/ ACGGCA (2)
AC (73)	GGT (48)	ATGA/CTGC (5)	AAAGA/AAAGC/ AACAC (1)	AAAGGG/AACAGC/ AACGGA (1)

Supplementary Table 12. List of GHs unique in *A. rabiei* among the selected most closely related *Dothideomycetes* fungi.

**S. No.**	**Gene ID**	**GH class**
1.	ST47_g262	GH38
2.	ST47_g763	GH38
3.	ST47_g963	GH76
4.	ST47_g1018	GH38
5.	ST47_g1025	GH38
6.	ST47_g1118	GH3
7.	ST47_g1126	GH3
8.	ST47_g1174	GH53
9.	ST47_g1198	GH16
10.	ST47_g1253	GH39
11.	ST47_g1280	GH17
12.	ST47_g1302	GH76
13.	ST47_g1305	GH23
14.	ST47_g1316	GH103
15.	ST47_g1324	GH16
16.	ST47_g1335	GH16
17.	ST47_g1339	GH92
18.	ST47_g1594	GH28
19.	ST47_g2370	GH78
20.	ST47_g3166	GH43
21.	ST47_g4719	GH71
22.	ST47_g4764	GH71
23.	ST47_g4979	GH76
24.	ST47_g4999	GH71
25.	ST47_g5365	GH5
26.	ST47_g5786	GH130
27.	ST47_g5821	GH3
28.	ST47_g5867	GH92
29.	ST47_g5868	GH75
30.	ST47_g5871	GH28
31.	ST47_g5910	GH1
32.	ST47_g6255	GH28
33.	ST47_g6429	GH3
34.	ST47_g6465	GH92
35.	ST47_g6471	GH10

36.	ST47_g7065	GH35
37.	ST47_g7212	GH20
38.	ST47_g7631	GH28
39.	ST47_g7698	GH76
40.	ST47_g7713	GH18
41.	ST47_g7849	GH89
42.	ST47_g7853	GH26
43.	ST47_g7966	GH109
44.	ST47_g8481	GH13
45.	ST47_g8635	GH1
46.	ST47_g9244	GH92
47.	ST47_g9444	GH76
48.	ST47_g9629	GH92
49.	ST47_g9633	GH109
50.	ST47_g9765	GH92
51.	ST47_g10073	GH10
52.	ST47_g10239	GH76
53.	ST47_g10579	GH16

Supplementary Table 13. Summary counts of CAZymes content present in 1,458 shared orthologous genes families among the necrotrophic fungi *A. rabiei*, *C. heterostrophus* and *P. tritici-repentis*.

CAZymes categories and their numbers					
**GH**	**GT**	**CE**	**PL**	**CBM**	**AA**
GH1 – 2	GT1 – 2	CE1 – 2	PL4 – 2	CBM1 – 2	AA3 – 8
GH2 – 1	GT2 – 6	CE3 – 1		CBM2 – 1	AA6 – 2
GH3 – 3	GT4 – 10	CE4 – 1		CBM3 – 1	AA7 – 1
GH4 – 2	GT22 – 1	CE8 – 1		CBM5 – 2	
GH5 – 5	GT25 – 3	CE9 – 3		CBM6 – 1	
GH9 – 2	GT31 – 2	CE10 – 3		CBM10 – 1	
GH10 – 4	GT32 – 2	CE11 – 2		CBM12 – 3	
GH13 – 1	GT34 – 10	CE12 – 4		CBM13 – 6	
GH15 – 1	GT41 – 2	CE14 – 1		CBM18 – 1	
GH16 – 1	GT48 – 103	CE15 – 1		CBM20 – 2	
GH17 – 2	GT55 – 2	CE16 – 2		CBM26 – 1	
GH18 – 9	GT87 – 3			CBM32 – 1	
GH20 – 3	GT90 – 1			CBM35 – 4	

GH24 – 1				CBM50 – 4	
GH27 – 3				CBM63 – 1	
GH28 – 3					
GH30 – 1					
GH36 – 1					
GH43 – 1					
GH55 – 2					
GH65 – 3					
GH76 – 11					
GH79 – 2					
GH88 – 1					
GH92 – 8					
GH95 – 2					
GH105 – 4					
GH109 – 3					
GH131 – 2					
**Total**					
**84**	**147**	**21**	**2**	**31**	**11**

Supplementary Table 14. Summary counts of CAZymes content present in 112 shared orthologous genes families among the biotrophic fungi *C. purpurea*, *B. graminis* f.sp. *tritici* and *B. graminis* f.sp. *hordei*.

**CAZymes categories and their numbers**					
**GH**	**GT**	**CE**	**PL**	**CBM**	**AA**
GH32 – 2	GT31 – 2	CE3 – 1	**-**	CBM1 – 1	AA5 – 1
	GT4 – 2	CE11 – 1		CBM13 – 1	
	GT90 – 1			CBM50 – 1	
**Total**					
**2**	**5**	**2**	**-**	**3**	**1**

Supplementary Table 15. Genes encoding transporters in *A. rabiei*.

**Superfamilies**	**TC No.**	**Subclass or subfamily description**	**Number**
Channels/Pores	1.A	Alpha -Type channels	55
	1.B	Beta-Barrel porins	5
	1.C	Pore-forming toxins (proteins and peptides)	3
	1.F	Vesicle Fusion Pores	5
	1.G	Viral Fusion Pores	1
Electrochemical potential-driven transporters	2.A	Porters (uniporters, symporters, antiporters)	**368**
	2.A.1	The Major Facilitator Superfamily (MFS)	165
	2.A.2	The Glycoside-Pentoside-Hexuronide (GPH): Cation Symporter Family	4
	2.A.3	The Amino Acid-Polyamine-Organocation (APC) Superfamily	32
	2.A.4	The Cation Diffusion Facilitator (CDF) Family	7
	2.A.5	The Zinc (Zn^{2+})-Iron (Fe^{2+}) Permease (ZIP) Family	6
	2.A.6	The Resistance-Nodulation-Cell Division (RND) Superfamily	4
	2.A.7	The Drug/Metabolite Transporter (DMT) Superfamily	11
	2.A.9	The Cytochrome Oxidase Biogenesis (Oxa1) Family	2
	2.A.16	The Tellurite-resistance/Dicarboxylate Transporter (TDT) Family	5
	2.A.17	The Proton-dependent Oligopeptide Transporter (POT) Family	10
	2.A.18	The Amino Acid/Auxin Permease (AAAP) Family	11
	2.A.19	The Ca^{2+}:Cation Antiporter (CaCA) Family	8

	2.A.20	The Inorganic Phosphate Transporter (PiT) Family	3
	2.A.21	The Solute:Sodium Symporter (SSS) Family	7
	2.A.22	The Neurotransmitter:Sodium Symporter (NSS) Family	1
	2.A.29	The Mitochondrial Carrier (MC) Family	32
	2.A.30	The Cation-Chloride Cotransporter (CCC) Family	1
	2.A.31	The Anion Exchanger (AE) Family	2
	2.A.36	The Monovalent Cation:Proton Antiporter-1 (CPA1) Family	4
	2.A.37	The Monovalent Cation:Proton Antiporter-2 (CPA2) Family	1
	2.A.38	The K^+ Transporter (Trk) Family	4
	2.A.39	The Nucleobase:Cation Symporter-1 (NCS1) Family	9
	2.A.40	The Nucleobase:Cation Symporter-2 (NCS2) Family	1
	2.A.41	The Concentrative Nucleoside Transporter (CNT) Family	1
	2.A.43	The Lysosomal Cystine Transporter (LCT) Family	2
	2.A.47	The Divalent Anion:Na^+ Symporter (DASS) Family	1
	2.A.49	The Chloride Carrier/Channel (ClC) Family	4
	2.A.50	The Glycerol Uptake (GUP) Family	1
	2.A.51	The Chromate Ion Transporter (CHR) Family	1
	2.A.52	The Ni^{2+}-Co^{2+} Transporter (NiCoT) Family	2
	2.A.53	The Sulfate Permease (SulP) Family	6
	2.A.54	The Mitochondrial Tricarboxylate Carrier (MTC) Family	1
	2.A.59	The Arsenical Resistance-3 (ACR3) Family	1
	2.A.66	The Multidrug/Oligosaccharidyl-	5

		lipid/Polysaccharide (MOP) Flippase Superfamily	
	2.A.67	The Oligopeptide Transporter (OPT) Family	3
	2.A.72	The K^+ Uptake Permease (KUP) Family	2
	2.A.78	The Branched Chain Amino Acid Exporter (LIV-E) Family	2
	2.A.89	The Vacuolar Iron Transporter (VIT) Family	1
	2.A.94	The Phosphate Permease (Pho1) Family	1
	2.A.96	The YaaH (YaaH) Family	3
	2.A.97	The Mitochondrial Inner Membrane K^+/H^+ and Ca^{2+}/H^+ Exchanger (LetM1) Family	1
Primary active transporters	3.A	P–P-bond-hydrolysis-driven transporters	**219**
	3.A.1	The ATP-binding Cassette (ABC) Superfamily	52
	3.A.2	The H^+- or Na^+-translocating F-type, V-type and A-type ATPase (F-ATPase) Superfamily	22
	3.A.3	The P-type ATPase (P-ATPase) Superfamily	18
	3.A.5	The General Secretory Pathway (Sec) Family	17
	3.A.7	The Type IV (Conjugal DNA-Protein Transfer or VirB) Secretory Pathway (IVSP) Family	1
	3.A.8	The Mitochondrial Protein Translocase (MPT) Family	20
	3.A.9	The Chloroplast Envelope Protein Translocase (CEPT or Tic-Toc) Family	8
	3.A.16	The Endoplasmic Reticular Retrotranslocon (ER-RT) Family	19
	3.A.18	The Nuclear mRNA Exporter (mRNA-E) Family	35
	3.A.19	The TMS Recognition/Insertion Complex (TRC) Family	1
	3.A.20	The Peroxisomal Protein Importer (PPI)	26

		Family	
	3.B	Decarboxylation-driven transporters	5
	3.D	Oxidoreduction-driven transporters	39
	3.E	Light absorption-driven transporters	2
Group translocators	4.C	Acyl CoA ligase-coupled transporters	**18**
	4.C.1	The Proposed Fatty Acid Group Translocation (FAT) Family	17
	4.C.2	The Carnitine O-Acyl Transferase (CrAT) Family	1
Transmembrane electron carriers	5.A	Transmembrane two-electron transfer carriers	3
	5.B	Transmembrane one-electron transfer carriers	2
Accessory electron carriers	8.A	Auxiliary transport proteins	**26**
	8.A.5	The Voltage-gated K+ Channel β-subunit (Kvβ) Family	6
	8.A.11	The Immunophilin-like Prolyl:Peptidyl Isomerase Regulator (I-PPI) Family	1
	8.A.13	The Tetratricopeptide Repeat (Tpr1) Family	1
	8.A.21	The Stomatin/Podocin/Band 7/Nephrosis.2/SPFH (Stomatin) Family	2
	8.A.27	The CDC50 P-type ATPase Lipid Flippase β-Subunit (CDC50) Family	1
	8.A.28	The Ankyrin (Ankyrin) Family	8
	8.A.30	The Nedd4-Family Interacting Protein-2 (Nedd4) Family	2
	8.A.34	The Endophilin (Endophilin) Family	5
Incompletely characterized transporters	9.A	Recognized transporters of unknown biochemical mechanism	**44**
	9.A.1	The Non ABC Multidrug Exporter (N-MDE) Family	3

	9.A.2	The Endomembrane protein-70 (EMP70) Family	1
	9.A.6	The ATP Exporter (ATP-E) Family	1
	9.A.8	The Ferrous Iron Uptake (FeoB) Family	1
	9.A.10	The Oligomeric Probable Pore-forming SpoIIA Toxin (SpoIIA) Family	6
	9.A.14	The G-protein-coupled receptor (GPCR) Family	6
	9.A.25	The Por Protein Secretin System (PorSS) Family	1
	9.A.26	The Lipid-translocating Exporter (LTE) Family	7
	9.A.27	The Non-Classical Protein Exporter (NCPE) Family	1
	9.A.34	Unclassified ClpB/EvpH protein	3
	9.A.36	The Ca^{2+}-dependent Phospholipid Scramblase (Scramblase) Family	1
	9.A.48	The Unconventional Protein Secretion (UPS) System	2
	9.A.49	Multi-pass membrane protein	1
	9.A.50	The Nuclear t-RNA Exporter (t-Exporter) Family	9
	9.A.54	The Lysosomal Cobalamin (B12) Transporter (L-B12T) Family	1
	9.B	Putative uncharacterized transport proteins	25
	9.C	Functionally characterized transporters lacking identified sequences	1
**TOTAL**			**821**

Supplementary Table 16. Genes involved in putative biosynthetic gene clusters in *A. rabiei*.

Gene Cluster	Type	Putative Biosynthetic Genes	Domain/Function	Scaffold	Location
Cluster 1	Terpene	ST47_g210	Intra molecular transferase activity	Scaffold 7	93538-95919
Cluster 2	Cf fatty acid	ST47_g984	Beta-ketoacyl-synthase	Scaffold 78	4127-9819
		ST47_g985	Malonyl CoA-acyl carrier protein transacylase		11388-17690
Cluster 3	Nrps-t1pks	ST47_g1537	Cytochrome P450	Scaffold 115	70900-74460
		ST47_g1538	O-methyltransferase		74903-7625
		ST47_g1539	Crotonyl Co-A reductase /alcohol dehydrogenase		77261-78511
		ST47_g1540	Beta-ketoacyl synthase		80987-93520
Cluster 4	Nrps	ST47_g1690	FAD dependent oxidoreductase	Scaffold 122	85491-86966
		ST47_g1693	AMP-dependent synthetase and ligase		92383-108664
		ST47_g1695	Lysine/ornithine N-monooxygenase		115556-117252
Cluster 5	Terpene	ST47_g2205	Polyprenyl synthetase	Scaffold 148	8840-10190
Cluster 6	T3pks	ST47_g2281	Chalcone and stilbene synthase domain protein	Scaffold 148	237024-238405
		ST47_g2284	Beta-lactamase		244246-248762
Clsuter 7	Nrps	ST47_g2791	Riboflavin biosynthesis protein RibD	Scaffold 161	24991-25560
		ST47_g2799	Aminotransferase		35983-37596
		ST47_g2802	AMP-dependent synthetase and ligase		42505-48413
		ST47_g2805	Lysine/ornithine N-monooxygenase		55194-56468
		ST47_g2806	Putative siderophore biosynthesis protein		57022-61789
		ST47_g2807	FAD dependent		64160-65809

			oxidoreductase		
Cluster 8	Terpene	ST47_g3203	Tryptophan dimethyl allytransferase activity	Scaffold 168	76380-77840
		ST47_g3205	Alpha/beta hydrolase fold protein		82829-84509
Cluster 9	T1pks	ST47_g3825	Aminotransferase class V	Scaffold 189	5021-6414
		ST47_g3826	AMP-dependent synthetase and ligase		7193-11313
		ST47_g3828	Beta-ketoacyl synthase		14932-22986
		ST47_g3829	O-methyltransferase		24291-25780
Cluster 10	Other	ST47_g4003	FAD linked oxidase domain protein	Scaffold 197	139-2015
		ST47_g4004	Dehydrogenase		2761-3871
		ST47_g4005	AMP-dependent synthetase and ligase		5032-8230
		ST47_g4008	Phenylalanine-specific permease		19918-22026
Cluster 11	Other	ST47_g5404	Pyruvate oxidase/decarboxylase	Scaffold 241	38532-40456
		ST47_g5410	AMP-dependent synthetase and ligase		52903-56190
Cluster 12	Terpene	ST47_g5562	Geranylgeranyl-diphosphate geranylgeranyl transferase activity	Scaffold 245	48950-50852
Cluster 13	Other	ST47_g5739	GCN5-related N-acetyltranferase	Scaffold 246	364011-364868
		ST47_g5740	NAD-dependent epimerase/dehydratase		365390-36615
		ST47_g5743	AMP-dependent synthetase and ligase		378045-381288
Clsuter 14	T1pks	ST47_g6273	Metallo-beta-lactamase family protein	Scaffold 258	310530-311642
		ST47_g6275	Beta-ketoacyl synthase		314565-321085
		ST47_g6277	Short-chain dehydrogenase/ reductase SDR		339615-340523
Cluster 15	T1pks	ST47_g6515	Beta-ketoacyl synthase	Scaffold 265	3479-11150
		ST47_g6516	Beta-ketoacyl synthase		11854-19735

		ST47_g6517	Crotonyl Co-A reductase / alcohol dehydrogenase		20099-21211
		ST47_g6518	NAD-dependent epimerase/dehydratase		21736-22814
Cluster 16	Other	ST47_g7073	AMP-dependent synthetase and ligase	Scaffold 286	104445-108296
		ST47_g7080	Phenylalanine-specific permease		122285-124256
Cluster 17	T1pks	ST47_g7183	Short-chain dehydrogenase/ reductase SDR	Scaffold 287	27211-27954
		ST47_g7185	Crotonyl Co-A reductase / alcohol dehydrogenase		30542-31869
		ST47_g7187	Beta-lactamase		34390-36349
		ST47_g7190	Beta-ketoacyl synthase		44122-51346
		ST47_g7195	Cytochrome P450		65485-67326
Cluster 18	T1pks	ST47_g7695	FAD linked oxidase domain protein	Scaffold 301	2057-3882
		ST47_g7696	Short-chain dehydrogenase/ reductase SDR		7345-8522
		ST47_g7697	Cytochrome P450		8570-10366
		ST47_g7698	Crotonyl Co-A reductase / alcohol dehydrogenase		10675-11868
		ST47_g7699	Cytochrome P450		12228-13958
		ST47_g7700	Beta-ketoacyl synthase		14689-23522
Cluster 19	T1pks	ST47_g8044	O-methyltransferase	Scaffold 314	3217-4633
		ST47_g8045	Short-chain dehydrogenase/ reductase SDR		5379-6419
		ST47_g8046	O-methyltransferase		6959-8330
		ST47_g8047	Beta-ketoacyl synthase		9688-16699
		ST47_g8049	Cytochrome P450		20656-22266
		ST47_g8051	Crotonyl Co-A reductase / alcohol dehydrogenase		24090-25115

Cluster 20	T1pks	ST47_g8065	Beta-ketoacyl synthase	Scaffold 315	28876-37239
Clsuter 21	T1pks	ST47_g8486	Phenylalanine-specific permease	Scaffold 327	240971-242706
		ST47_g8492	Beta-ketoacyl synthase		257757-265846
		ST47_g8494	Crotonyl Co-A reductase / alcohol dehydrogenase		267346-268427
		ST47_g8495	Short-chain dehydrogenase/ reductase SDR		269155-270275
		ST47_g8498	Short-chain dehydrogenase/ reductase SDR		276019-278998
		ST47_g8499	Beta-ketoacyl synthase		279585-286398
		ST47_g8500	3-hydroxybutyryl-CoA dehydrogenase		287651-289450
Cluster 22	Other	ST47_g8723	AMP-dependent synthetase and ligase	Scaffold 331	118471-121704
		ST47_g8726	Cytochrome P450		129659-133547
Cluster 23	T1pks	ST47_g8726	Cytochrome P450	Scaffold 331	129659-133547
		ST47_g8732	Beta-ketoacyl synthase		147182-152635
Cluster 24	Terpene	ST47_g9109	Farnesyl-diphosphate Farnesyltransferase activity	Scaffold 336	540093-541743
Cluster 25	Cf fatty acid	ST47_g9884	Alpha/beta hydrolase domain-containing protein	Scaffold 358	34451-35722
		ST47_g9885	Alpha/beta hydrolase fold protein		36284-42661
		ST47_g9886	Beta-ketoacyl synthase		43248-44872
		ST47_g9891	Short-chain dehydrogenase/ reductase SDR		49560-50237
		ST47_g9893	Isochorismatase		52308-53687
Clsuter 26	Other	ST47_g9915	Short-chain dehydrogenase/ reductase SDR	Scaffold 363	18895-19809
		ST47_g9918	AMP-dependent synthetase and ligase		27020-30622

		ST47_g9921	Alcohol dehydrogenase		39854-40995
		ST47_g9926	Argininosuccinate lyase/adenylosuccinate lyase		47692-49172

Supplementary Table 17. Summary counts of CAZymes content of *A. rabiei*.

**CAZymes categories and their numbers**					
**GH**	**GT**	**CE**	**PL**	**CBM**	**AA**
GH1 – 12	GT1 – 14	**CE1 – 12**	**PL1 – 10**	**CBM1 – 8**	**AA1 – 10**
GH2 – 8	**GT2 – 87**	**CE4 – 17**	**PL3 – 10**	CBM2 – 4	AA2 – 9
**GH3 – 29**	GT3 – 1	**CE5 – 12**	PL4 – 3	CBM6 – 3	**AA3 – 60**
GH5 – 13	**GT4 – 70**	CE8 – 4		CBM12 – 4	AA4 – 7
GH6 – 3	GT5 – 2	**CE9 – 13**		**CBM13 – 17**	AA5 – 1
GH7 – 3	GT8 – 4	**CE10 – 71**		CBM20 – 4	**AA6 – 11**
GH9 – 2	GT9 – 2	**CE11 – 58**		CBM21 – 6	**AA7 – 30**
GH10 – 18	GT15 – 7	CE14 – 6		CBM22 – 1	**AA9 – 21**
GH11 – 3	GT20 – 7			CBM26 – 7	AA10 – 8
GH12 – 6	GT22 – 8			**CBM32 – 8**	
GH13 – 9	GT24 – 1			CBM35 – 3	
GH15 – 3	GT25 – 4			CBM42 – 1	
GH16 – 13	GT26 – 1			CBM43 – 2	
GH17 – 11	GT28 – 7			**CBM48 – 24**	
**GH18 – 33**	GT30 – 3			**CBM50 – 25**	
GH20 – 7	GT31 – 9			CBM51 – 3	
GH23 – 9	GT32 – 4			CBM54 – 2	
GH26 – 1	**GT34 – 79**			CBM57 – 4	
GH27 – 7	GT35 – 1				
**GH28 – 36**	GT39 – 4				
GH29 – 1	**GT41 – 20**				
GH31 – 11	GT47 – 1				
GH 32 – 22	**GT48 – 315**				
GH33 – 7	GT49 – 2				
GH35 – 5	GT50 – 7				
GH 36 – 1	GT51 – 5				
GH37 – 2	GT54 – 1				
GH38 – 5	GT55 – 14				
GH39 – 2	GT57 – 2				

GH43 – 17	GT58 – 2				
GH45 – 2	GT59 – 1				
GH47 – 13	GT60 – 1				
GH51 – 2	GT62 – 3				
GH53 – 3	GT66 – 13				
GH54 – 1	GT69 – 2				
GH55 – 5	GT71 – 1				
GH62 – 2	GT76 – 1				
GH63 – 1	GT83 – 3				
GH65 – 20	GT87 – 12				
GH71 – 4	GT90 – 15				
GH72 – 18					
GH75 – 2					
**GH76 – 60**					
GH78 – 6					
GH81 – 2					
GH84 – 8					
GH85 – 3					
GH89 – 1					
**GH92 – 50**					
GH94 – 1					
GH99 – 1					
GH103 – 4					
GH109 – 12					
GH125 – 3					
GH127 – 1					
GH128 – 5					
GH130 – 10					
GH132 - 7					
**Total**					
**546**	**736**	**193**	**23**	**126**	**157**

Supplementary Table 18. Summary of proteins putatively involved in pathogen-host interactions in *A. rabiei*.

S.No.	Mutant phenotype	Number of proteins
1.	Loss of pathogenicity	222
2.	Reduced virulence	1,037
3.	Mixed outcome	172
4.	Effectors	13
	**Total**	**1,444**
5.	Unaffected pathogenicity	1,073
6.	Increased virulence (Hypervirulence)	58
7.	Lethal	93
8.	Sensitive to chemicals	15
9.	Resistant to chemicals	5
10.	Partially lost Ax21	16
11.	Enhanced antagonism	1
12.	Wild type mutualism	1
13.	Essential	1

Supplementary Table 19. Summary counts of CAZymes content of *A. rabiei* secretome.

**CAZymes categories and their numbers**					
**GH**	**GT**	**CE**	**PL**	**CBM**	**AA**
GH1 – 1	**GT2 – 2**	**CE1 – 2**	**PL1 – 8**	**CBM1 – 5**	**AA1 – 5**
GH2 – 2	**GT4 – 1**	**CE4 – 3**	**PL3 – 8**	**CBM13 – 4**	**AA2 – 3**
**GH3 – 8**		**CE5 – 9**	**PL4 – 3**	**CBM20 – 3**	**AA3 – 4**
**GH5 – 6**		**CE8 – 2**		CBM42 – 1	AA5 – 1
GH6 – 3		**CE10 – 16**		CBM48 – 1	**AA7 – 9**
GH7 – 3				**CBM50 – 10**	**AA9 – 17**
GH10 – 5					
GH11 – 3					
GH12 – 3					
GH13 – 1					
GH15 – 1					
GH16 – 2					
GH17 – 2					
**GH18 – 6**					

GH26 – 1					
**GH28 – 10**					
GH29 – 1					
GH31 – 2					
GH32 – 3					
GH35 – 4					
**GH43 – 6**					
GH45 – 1					
GH47 – 2					
GH51 – 1					
GH53 – 1					
GH54 – 1					
GH62 – 2					
GH65 – 1					
GH71 – 2					
GH76 – 1					
GH78 – 2					
GH89 – 1					
GH92 – 2					
GH103 – 1					
GH127 – 1					
GH128 – 3					
**Total**					
**95**	**3**	**32**	**19**	**24**	**39**

Supplementary Table 20. The effector proteins putatively involved in pathogen-host interactions in *A. rabiei*.

S.No.	Mutant phenotype	Number of proteins
1.	Loss of pathogenicity	12
2.	Reduced virulence	53
3.	Mixed outcome	2
4.	Effectors	3
	**Total**	**70**
5.	Unaffected pathogenicity	95
6.	Increased virulence	2

Supplementary Table 21. The unannotated secretory proteins having tandem repeats.

S.No.	Gene ID	Repeat Length	Repeat Frequency	Position	Repeat Sequence
1.	ST47_g587	3	10	51-80	GAA
2.	ST47_g917	6<br>9	19<br>11	217-316<br>693-775	DAAPV-<br>PTSTPDE--
3.	ST47_g972	13	14	343-518	LGLPAGGLPTLPN
4.	ST47_g1978	2	14	181-209	GN
5.	ST47_g2073	1	16	88-103	G
6.	ST47_g2119	2	12	25-48	GT
7.	ST47_g3121	10	12	295-410	DPYPTETPEY
8.	ST47_g3860	1	10	203-212	G
9.	ST47_g4696	6	11	45-111	SSTASL-
10.	ST47_g4774	2	13	44-69	HD
11.	ST47_g5062	8	10	123-202	SAALVNFP
12.	ST47_g5065	1	11	58-68	D
13.	ST47_g6278	5	12	335-394	TGNTN
14.	ST47_g6554	10<br>10	10<br>22	162-263<br>318-550	GGLL---PTDIL---<br>TDVLGDLATGLP
15.	ST47_g6845	5<br>6	17<br>17	728-813<br>496-587	LPIPD<br>LPDAG-
16.	ST47_g7300	2	11	59-80	DT
17.	ST47_g7602	6	15	316-400	QQSANG
18.	ST47_g7675	2	12	140-164	KG
		6	25	297-446	QTGTGA
19.	ST47_g8191	2	15	198-228	GK
20.	ST47_g8756	2	9	104-122	GK
21.	ST47_g9151	5	12	261-314	AAATS

Supplementary Table 22. The prediction of *in planta* localization of mature effector proteins.

S.No.	Cell organelle	Number of effector proteins
1.	Cytoplasm	72
2.	Extracellular	448
3.	Nucleus	164
4.	Mitochondria	14
5.	Chloroplast	30
6.	Peroxisome	2
7.	Lysosome	8
8.	Plasma membrane	10
9.	Vacuole	10

Supplementary Table 23. The prediction of NLS in effectors predicted to localize in nucleus inside the host.

S.No.	Effector protein	Predicted NLS
1.	ST47_g880	196 - RMRRSKKLSQKDKPPKERRRRRKRP - 220
2.	ST47_g1734	22 - RKRRRRR - 28
3.	ST47_g2418	461 - KRNSKVFSARRPGKRV - 476
4.	ST47_g3617	98 - KPRRALP - 104<br>113 - RRRPTTRTK - 121
5.	ST47_g3668	281 - KRAIAYKLKKRMELSR - 296
6.	ST47_g4241	231 - GGRKYAKEKRSHSEEGRPHRHRDAYREGREEGRDDR EHGRHRHRGAYREGKEDRRRDRAR - 290
7.	ST47_g4988	60 - RRRLHRRRRRPCSARSLHRP - 79
8.	ST47_g6278	510 - AGKPGAPAKPERRQSSEK - 527
9.	ST47_g7291	301 - RGRARDRGFGNGDANGSRRNSPSGPAPPLQNPRKRRR EEKRQEKRR - 346
10.	ST47_g7442	111 - PRRGRGGRGFRGRGFRGRGRGGR - 133
11.	ST47_g7463	14 - KA - 15
12.	ST47_g7675	112 - GGKAAKGAKGKGKGKGKGKGKGKGKGK - 138

13.	ST47_g8330	83 - KR - 84<br>108 - GSGKSKKKGKGGAG - 121
14.	ST47_g8561	350 - ARR - 352
15.	ST47_g9775	381 - GGRGGRGGNGRGNN - 394
16.	ST47_g9812	123 - KSRRGWKGKRKTKR - 136
17.	ST47_g10316	143 - PRTKAKNKRNRDKYKAKKRDERHKREQARKK - 173
18.	ST47_g10372	16 - KQARRSRSAKASAKRERSKSKGRKEAGKEAGKEAGKE AGKEAGKESR - 62

Supplementary Table 24. The list of primers used in this study.

No.	Name	Sequence (5′ to 3′)	Purpose
1.	Com1	GCATGCCATATCGCCAGT	For determining mating type 1 and 2 of *A. rabiei*
2.	SP21	ACAGTGAGCCTGCACAGTTC	For determining mating type 1 of *A. rabiei*
3.	Tail5	CGCTATTTTATCCAAGACAC ACC	For determining mating type 2 of *A. rabiei*
